# Experiences of and barriers to transition-related healthcare among Korean transgender adults: focus on gender identity disorder diagnosis, hormone therapy, and sex reassignment surgery

**DOI:** 10.4178/epih.e2018005

**Published:** 2018-02-27

**Authors:** Hyemin Lee, Jooyoung Park, Bokyoung Choi, Horim Yi, Seung-Sup Kim

**Affiliations:** 1 Department of Public Health Sciences, Graduate School of Korea University, Seoul, Korea; 2 Department of Social and Behavioral Sciences, Harvard T. H. Chan School of Public Health, Boston, MA, USA

**Keywords:** Transgender persons, Health services for transgender persons, Sex reassignment surgery, Sexual and gender minorities, Gender identity, Republic of Korea

## Abstract

**OBJECTIVES:**

Transgender people may encounter barriers to transition-related healthcare services. This study aimed to investigate the experiences of transition-related healthcare and barriers to those procedures among transgender adults in Korea.

**METHODS:**

In 2017, we conducted a nationwide cross-sectional survey of 278 transgender adults, which named Rainbow Connection Project II, in Korea. We assessed the prevalence of transition-related healthcare, including gender identity disorder (GID) diagnosis, hormone therapy, and sex reassignment surgery. To understand the barriers to those procedures, we also asked participants for their reasons for not receiving each procedure. Further, this study examined their experiences of and the reasons for using non-prescribed hormone medications.

**RESULTS:**

Of transgender people participated in the survey, 91.0% (n=253/278) were diagnosed with GID, 88.0% (n=243/276) received hormone therapy, and 42.4% (n=115/271) have had any kind of sex reassignment surgery. Cost was the most common barrier to transition-related healthcare among Korean transgender adults. Other common barriers were identified as follows: negative experiences in healthcare settings, lack of specialized healthcare professionals and facilities, and social stigma against transgender people. Among those who had taken hormone medications, 25.1% (n=61/243) reported that they had ever purchased them without a prescription.

**CONCLUSIONS:**

Our findings suggest that barriers to transition-related healthcare exist in Korea and constrain transgender individuals’ safe access to the needed healthcare. Institutional interventions are strongly recommended to improve access to transition-related healthcare. These interventions include provision of programs to train Korean healthcare professionals and expansion of national health insurance to include these procedures.

## INTRODUCTION

Transgender people have a gender identity or expression that is inconsistent with their legal sex at birth [1,2]. Transgender individuals can be classified as transwoman, transman, non-binary transgender person, etc. Non-binary transgender people refer to those who do not identify themselves as either man or woman [3]. Unlike transgender individuals, cisgender people have a gender identity or expression that matches their legal sex at birth [4].

In the US, prevalence of the transgender population was estimated to be 390 per 100,000 individuals [5]. This estimation is based on a meta-analysis of 12 national surveys using three databases in 2017, so the results address problems in previous studies resulting from non-probability sampling and representativeness estimating the transgender population size. By extrapolating the estimated frequency of transgender individuals from the meta-analysis study to the total Korean population, which is 51,635,256 as of February 2018 [6], there are an estimated 201,377 transgender individuals in Korea.

Through gender transition, transgender people live their lives with the gender expression or presentation that affirms their gender identity [7]. When medically necessary, many transgender individuals undergo transition-related healthcare or medical transition, including gender identity disorder (GID) diagnosis, hormone therapy, and sex reassignment surgery [7]. Transition-related healthcare can alleviate gender dysphoria and improve physical and mental health as well as quality of life among transgender people [8-10]. Although not all transgender individuals receive hormone therapy or sex reassignment surgery [7,11], transition-related healthcare is a salient factor for transgender people’s health and wellbeing [4].

In Korea, access to transition-related healthcare is critical not only for reducing transgender individuals’ own gender dysphoria, but also for institutional factors, such as legal sex change and exemption from military service [7,11]. Legal sex change is based on “The Guidelines for the Handling of Petition for Legal Sex Change Permit of Transgender People” in Article 435 of the Supreme Court Family Relation Registration Regulation in Korea [12,13]. The regulation requires applicants to receive a psychiatric diagnosis of GID, remove reproductive capacity, and have sex reassignment surgery through investigative matters and compulsory documentation.

Further, all individuals whose legal sex is male and who have Korean nationality are obliged to serve in military services, as mandated by the Military Service Act and Article 39 (1) of the Constitution of Korea. This obligation also applies to transwomen who do not change their legal sex at birth [14,15]. Under such circumstances, transwomen who wish to be exempt from military service must obtain a GID diagnosis, receive hormone therapy, and even undergo sex reassignment surgery [11,14,15]. Transmen are also required to have a physical examination for military service after changing their legal sex to male [14].

However, the Korean healthcare system and policies related to transition-related healthcare are not well established [7,11]. Standard curricula for educating and training healthcare professionals do not include transition-related healthcare, and few training manuals or guidelines present this information. Further, transgender individuals are burdened with the entire cost of hormone therapy and sex reassignment surgery because those medical procedures are not covered by national health insurance.

Research on the experiences of and barriers to transition-related healthcare among transgender people is also lacking in Korea. Only 22 out of 128 studies on the health of lesbian, gay, bisexual, and transgender people that published before 2013 included trans2gender people, and most were case reports of transgender patients or clinical studies introducing methods for sex reassignment surgery [16]. Besides hospital-based clinical studies, research regarding the experiences of transition-related healthcare among transgender people is limited to only a single qualitative study in 2017 [11].

This study aimed to investigate the experiences of and barriers to transition-related healthcare, including GID diagnosis, hormone therapy, and sex reassignment surgery, for transgender adults in Korea. In addition, this study examined the experiences of non-prescribed hormone use and the reasons for that use to identify unsafe practices regarding transition-related healthcare among Korean transgender people.

## MATERIALS AND METHODS

### Data and study population

This study conducted a nationwide cross-sectional survey of Korean transgender adults to explore their experiences of transition-related healthcare and the barriers to those procedures. Participants were ≥19 years old and self-identified as either: 1) transwoman, 2) transman, or 3) non-binary transgender person with the experiences of transition-related healthcare. Because non-binary transgender people also undergo transition-related healthcare based on institutional factors as well as medical treatments for relieving gender dysphoria [11], they were also included in the study.

Data were collected through an online-based survey from June 27 to August 31, 2017. To advertise the survey and recruit study participants, this study utilized Korea Queer Festivals held in Seoul and Daegu, four healthcare institutions in Seoul that provide transition-related healthcare for transgender patients, and three online/offline transgender communities. Informed consent for participating in the survey and using the data for academic purposes was collected from all respondents. As compensation for participation, all respondents were rewarded 10,000 Korean won (KRW). This study protocol was approved by the institutional review board of Korea University (no. 1040548-KU-IRB-17-67-A-1).

A total of 347 transgender adults participated in the survey. Among them, there were 5 respondents who did not consent to academic use of the study, 62 respondents who did not provide demographic information including age and gender identity, and 2 respondents who did not respond to the question about GID diagnosis. After excluding those respondents, the final study population consisted of 278 transgender individuals.

### Measurement

Questionnaires were used to collect information on the experiences of transition-related healthcare, access to healthcare, health status, etc. Participants answered approximately 160-230 questions, including several additional questions depending on their gender identity and experiences of transition-related healthcare.

Regarding respondents’ gender identity, this study assessed both legal sex at birth and current gender identity to classify those with mismatching legal sex at birth and current gender identity as transgender people and those with matching identities as cisgender people (Table 1) [17]. Among transgender respondents, those whose legal sex at birth was male and currently identifying as woman were classified as transwomen, whereas those whose legal sex at birth was female and currently identifying as man were classified as transmen. Respondents who identified as neither man nor woman were categorized as non-binary transgender people. In this study, non-binary transgender individuals and transwomen whose legal sex at birth were male were classified as transfeminine, whereas non-binary transgender individuals and transmen whose legal sex at birth were female were classified as transmasculine [4].

Age, sexual orientation, and residential area were collected as demographic characteristics. Age was dichotomized as 19-29 years or 30-50 years. Respondents’ sexual orientation was classified as homosexual, bisexual, heterosexual, or asexual. Residential area was categorized into three categories (Seoul metropolitan city, other metropolitan cities, or other cities and counties). Respondents also provided information on socioeconomic status, such as educational level (≤high school graduate, college graduate, university graduate, or ≥graduate school graduate), annual household income (<10,000,000, 10,000,000-19,990,000, 20,000,000-29,990,000, 30,000,000-49,990,000, or ≥50,000,000 KRW), and employment status (unemployed, non-precarious employment, precarious employment, self-employed, or unpaid family worker). In addition, respondents reported the route to participating in the survey.

We classified transition-related healthcare into psychiatric diagnosis for GID, hormone therapy, and sex reassignment surgery. For GID diagnosis, respondents answered the question, “Have you ever received a psychiatric diagnosis for GID based on the Korean Standard Classification of Diseases?” Those who did not seek a GID diagnosis were asked to report the reasons why, and multiple choices could be selected.

Hormone therapy was measured with the question, “Are you currently receiving hormone therapy?” Response options were: “I am currently receiving hormone therapy”; “I received hormone therapy, but not currently”; “I never receive hormone therapy, but I want to (or plan to) sometime later”; “I am not sure whether I want to receive hormone therapy”; or “I do not want to receive hormone therapy”. Respondents who chose “I am currently receiving hormone therapy” or “I received hormone therapy, but not currently” were classified as those having experienced hormone therapy, and they were asked to respond to a follow-up question about purchasing non-prescribed hormone medications. For those who have purchased non-prescribed hormone medications, they were asked to report the route of access and the reasons why. In addition, respondents who did not receive hormone therapy were asked to identify the reasons why. Multiple responses could be selected for the questions regarding the route of and the reasons for purchasing hormone medications without a prescription and the reasons for not receiving hormone therapy.

Regarding sex reassignment surgery, respondents were asked whether they have had at least one surgical procedure related to transition. For those who did not have such surgery, additional questions were asked about the reasons why, and multiple choices could be selected.

This study also measured the costs of GID diagnosis and sex reassignment surgery. Regarding the cost of GID diagnosis, respondents chose a response from 0 to 1 million KRW. Since the total cost of hormone therapy varies with the duration of partaking in the therapy, an average cost was not presented. Sex reassignment surgery was classified into breast/chest surgery, genital removal surgery, genital reconstruction surgery, facial surgery, voice surgery (only for transfeminine respondents), and other surgeries. For the cost of sex reassignment surgery, respondents responded from 1 to 50 million KRW, with suggestions of the average cost and standard deviation. Additionally, respondents reported their experiences of delaying or avoiding hospital visits and being denied healthcare services within the past 12 months.

### Statistical analysis

This study analyzed the experiences and barriers that transgender people faced during transition-related healthcare with descriptive statistics, and the results were stratified by participants’ gender identity. All statistical analyses were performed using Stata/SE version 13.0 (StataCorp., College Station, TX, USA).

## RESULTS

### Socio-demographic characteristics and experiences of transition-related healthcare

Among the total study population, 173 (62.2%) were transfeminine and 105 (37.8%) were transmasculine. Of all respondents, 218 (78.4%) were 19-29 years and 60 (21.6%) were 30-50 years old, indicating that the majority were in their 20s (Table 2). Regarding sexual orientation and educational level, heterosexual (n=129/278, 46.4%) and university graduate (n=145/254, 57.1%) were the most frequent responses. Approximately half of respondents reported an annual household income of <10,000,000 (n=69/252, 27.4%) and 10,000,000-19,990,000 KRW (n=53/252, 21.0%). More than three-quarters of respondents were unemployed (n=118/253, 46.6%) or precarious employees (n=78/253, 30.8%), and a majority resided in metropolitan cities. Most respondents participated in the survey through four healthcare institutions (n=130/278, 46.8%) and online/offline transgender communities (n=126/278, 45.3%).

Among 278 transgender adults, 253 (91.0%) had been diagnosed with GID (Table 3). Meanwhile, 243 of 276 respondents (88.0%) who answered the question about hormone therapy were receiving or had previously received hormone therapy, and 115 of 271 respondents (42.4%) who answered the question about sex reassignment surgery have had at least one of transition-related surgery. The prevalence of a GID diagnosis was significantly higher in the transfeminine group (n=164/173, 94.8%) and 30-50-year-old group (n=59/60, 98.3%) than in the transmasculine group (n=89/105, 84.8%) and 19-29-year-old group (n=194/218, 89.0%). The prevalence of hormone therapy also was significantly higher in the transfeminine group (n=160/171, 93.6%) and 30-50-year-old group (n=58/59, 98.3%) than in the transmasculine group (n=83/105, 79.0%) and 19-29-year-old group (n=185/217, 85.3%). However, the prevalence of sex reassignment surgery was significantly higher in the transmasculine group (n=58/103, 56.3%) compared to the transfeminine group (n=57/168, 33.9%).

### Barriers to transition-related healthcare

A majority of transfeminine (n=164/173, 94.8%) and transmasculine (n=89/105, 84.8%) individuals reported that they were diagnosed with GID (Table 4). Among these 25 individuals who did not receive a GID diagnosis, 12 (48.0%) answered, “I was having financial difficulties”.

Among 276 respondents, 225 (81.5%) reported that they were currently receiving hormone therapy (Table 5). In terms of gender identity, 150 of 171 transfeminine (87.7%) and 75 of 105 transmasculine (71.4%) individuals were currently receiving hormone therapy. Those who were not currently receiving hormone therapy (n=51/276, 18.5%) were asked about the reasons for not receiving or discontinuing the therapy, and the most common response was “I was having financial difficulties” (n=28/51, 54.9%).

Among the 243 respondents who had ever received hormone therapy, 61 (25.1%) reported that they had ever purchased non-prescribed hormone medications (Table 6). Those who had purchased non-prescribed hormone medications were asked about the purchasing route of such medications, and the most common response was “over-the-counter medicines” (n=28/61, 45.9%), which is similar to contraceptives. With respect to reasons for purchasing non-prescribed hormone medications, the most common response was “I did not have a diagnosis from a psychiatrist” (n=34/61, 55.7%).

A total of 115 among 271 transgender respondents reported that they had undergone at least one kind of sex reassignment surgery, including 57 of 168 transfeminine (33.9%) and 58 of 103 transmasculine (56.3%) individuals (Table 7). Among 271 respondents, 156 (57.6%) did not have any kind of sex reassignment surgery. The most common reason why they did not have such surgery was “The surgery was too costly” (n=122/156, 78.2%).

## DISCUSSION

This study analyzed cross-sectional data of 278 transgender adults to investigate the experiences of and barriers to transition-related healthcare in Korea. The results suggest that 91.0% of surveyed transgender adults were diagnosed with GID, 88.0% had received hormone therapy, and 42.4% had undergone at least one kind of transition-related surgery. The primary reason for not accessing transition-related healthcare was due to the cost of such procedures. This study additionally analyzed the cost that participants paid for transition-related healthcare (Appendix 1). A majority (n=170, 67.7%) reported that they paid 250,000-490,000 KRW for a GID diagnosis. The average cost of each kind of sex reassignment surgery was calculated and classified by respondents’ gender identity. These results indicated that genital reconstruction surgery was the biggest financial burden for transgender respondents, estimated at an average of 15,148,000 KRW for transfeminine and 20,571,000 KRW for transmasculine individuals.

Along with the high costs of medical transition, prejudice and discrimination of healthcare professionals towards transgender individuals were primary factors limiting use of transition-related healthcare. This study further analyzed respondents’ experiences of delaying or avoiding hospital visits and being denied services by healthcare professionals (Appendix 2). Among 262 participants, 112 (42.7%) reported that they themselves had delayed or avoided hospital visits, and 14 (5.3%) reported that they were denied healthcare services within the past 12 months. According to a previous survey about discrimination based on sexual orientation and gender identity in Korea, 28 of 78 transgender respondents (35.9%) who visited healthcare institutions within the past 5 years reported that they experienced discrimination from healthcare professionals [18].

Another barrier to transition-related healthcare was based on the limited knowledge of healthcare professionals on medical transition and the lack of healthcare institutions that can provide such procedures. Among the 25 transgender participants who did not receive a GID diagnosis, 28.0% responded that “I could not find a psychiatrist who would provide an adequate diagnosis.” Since GID diagnosis is required to receive hormone therapy and sex reassignment surgery in the majority of cases, the diagnosis plays an essential role in determining access to medical transition [19]. Among respondents who did not receive hormone therapy, 33.3% reported a reason of “I did not have a medical certificate from a psychiatrist” and 15.7% reported a reason of “I did not have access to healthcare facilities that provided hormone therapy”.

The World Professional Association for Transgender Health has published “Standards of Care for the Health of Transsexual, Transgender, and Gender-Nonconforming People,” providing a clinical guideline for transition-related healthcare [2]. In North American and European countries, education and training programs on medical transition for healthcare professionals have been developed in healthcare institutions and academic societies [7]. Moreover, previous studies indicate that educating and training medical students about transition-related healthcare strengthens their competency as healthcare professionals with respect to knowledge, attitude, and skills regarding transgender health [20,21]. Since Korean medical schools do not educate about medical transition, healthcare professionals have relatively little knowledge about such procedures [7].

Negative social perception towards transgender people was another barrier that may inhibit these individuals from accessing transition-related healthcare. Participants provided the following reasons for not receiving hormone therapy: “I believed it would raise difficulties in my economic activities such as finding employment and working” (47.1%), “I was worried about potential stigma from other people around me” (25.5%), and “My family and/or friends advised against it” (15.7%). Those who did not have sex reassignment surgery also responded with the following reasons: “I believed it would raise difficulties in my economic activities such as finding employment and working” (36.5%), “My family and/or friends advised against it” (25.0%), and “I was worried about potential stigma from other people around me” (20.5%). Transgender individuals could experience difficulties in economic activities, such as getting a job or maintaining a career, due to medical transition experiences. Further, family members and friends of transgender individuals may oppose their medical transition.

Our results indicate that transgender people encountered barriers to accessing transition-related healthcare due to financial burden, negative experiences in healthcare settings, lack of specialized healthcare professionals and medical facilities, and social stigma against transgender individuals in Korea. These barriers could contribute to unsafe practices regarding transition-related healthcare [22,23]. In this study, 25.1% of 243 transgender participants reported purchasing non-prescribed hormone medications. They used over-the-counter medicines that contained hormonal components or purchased hormone drugs through domestic or foreign online vendors/suppliers or acquaintances/friends. For patient safety, healthcare professionals should control the dose of hormone therapy depending on the health condition of the transgender individual, and regular clinical follow-ups also are essential in hormone therapy. Therefore, self-prescribed hormone medications can be hazardous because the side effects, such as thromboembolism or elevated liver enzymes, may not be identified or appropriately treated [7,23].

To improve access to transition-related healthcare for transgender populations, national health insurance coverage should be expanded for such procedures. Among 118 nations, 45 countries pay for the costs of at least one kind of medical transition through national health insurance or public healthcare system [7]. Currently, the Korean public healthcare system does not cover the costs of hormone therapy or sex reassignment surgery for transgender people. Considering that expanding health insurance coverage could be an institutional intervention to relieve the financial burden of transition-related healthcare, more active discussions on this topic should be facilitated.

A noteworthy limitation of this study is that respondents may not accurately represent the whole transgender population in Korea. However, the population size is unknown because no studies have identified the transgender population size as of May 2018 in Korea [7,16]. We tried to recruit the maximum number of transgender individuals from healthcare institutions, Korea Queer Culture Festivals, and online/offline transgender communities through convenience sampling. Nonetheless, such data collection strategies may influence and potentially bias the study’s findings. Among 278 transgender respondents, 46.8% participated through healthcare institutions, whereas 45.3 and 7.9% became involved through online/offline transgender communities and the Korea Queer Culture Festivals, respectively. The number of respondents from healthcare institutions who had received a GID diagnosis or hormone therapy was relatively higher than that of respondents from online/offline transgender communities and the Korea Queer Culture Festivals. We cannot exclude the possibility that the experiences of respondents from healthcare institutions who received a GID diagnosis or hormone therapy may be overrepresented. In addition, the majority of respondents (78.4%) were in their 20s, which may be attributed to use of an online-based survey. Consequently, the findings of this study should be cautiously interpreted.

Despite these limitations, the present study also has key strengths. First, this study included the largest number of transgender participants in Korea as of May 2018, since all available methods was fully utilized to collect data. Second, this is the first academic research to identify the experiences of and barriers to transition-related healthcare of Korean transgender adults. Considering our findings, future research should continue to examine the health of the transgender population in Korea.

In conclusion, transition-related healthcare is medically necessary for transgender individuals to alleviate their gender dysphoria. Also, it is a legal prerequisite to change their gender identity on official documents and a method to protect themselves against social violence and discrimination and to access sex-segregated facilities, such as public toilets [19]. Therefore, expansions of health insurance coverage to include medical transition is needed to improve access to such medical procedures and support the health of transgender people. In addition, education and training programs for healthcare professionals should be institutionalized to ensure that they can provide appropriate transition-related healthcare services to transgender people. Beyond the healthcare setting, additional discussions are needed to improve social acceptance and recognition of transgender population and reduce stigma and discrimination against them. We strongly recommend that government-conducted nationwide surveys should include questions about respondents’ gender identity to promote future research on the health of transgender people. Lastly, based on our findings about barriers to transition-related healthcare, implementation of institutional intervention is urgently required to improve the health of Korean transgender individuals.

## Figures and Tables

**Table 1.** Gender identity measured with a two-step method^1^

Current gender identity	Legal sex at birth	
	Male	Female
Man	Cisgender^2^	Transmasculine
Woman	Transfeminine	Cisgender^2^
Do not identify as man or woman	Transfeminine	Transmasculine

1 Modified from Reisner et al. Lancet 2016;388:412-436 [4].

2 Cisgender defined as a non-transgender whose legal sex at birth is in accordance with their current gender identity.

**Table 2.** Distribution of study population and prevalence of gender identity spectrum by socio-demographic characteristics among transgender adults in Korea

	All respondents (n=278)	Gender identity spectrum^1^		p-value^2^
		Transfeminine (n=173)	Transmasculine (n=105)	
Age (yr)				<0.001
19-29	218 (78.4)	124 (56.9)	94 (43.1)	
30-50	60 (21.6)	49 (81.7)	11 (18.3)	
Sexual orientation				0.001
Heterosexual	129 (46.4)	65 (50.4)	64 (49.6)	
Homosexual	29 (10.4)	24 (82.8)	5 (17.2)	
Bisexual	96 (34.5)	66 (68.8)	30 (31.3)	
Asexual	24 (8.6)	18 (75.0)	6 (25.0)	
Educational level^3^				0.87
≤High school graduate	57 (22.4)	34 (59.7)	23 (40.4)	
College graduate	39 (15.4)	25 (64.1)	14 (35.9)	
University graduate	145 (57.1)	86 (59.3)	59 (40.7)	
≥Graduate school graduate	13 (5.1)	9 (69.2)	4 (30.8)	
Annual household income (10^4^ KRW)				0.24
<1,000	69 (27.4)	48 (69.6)	21 (30.4)	
1,000-1,999	53 (21.0)	30 (56.6)	23 (43.4)	
2,000-2,999	45 (17.9)	30 (66.7)	15 (33.3)	
3,000-4,999	43 (17.1)	25 (58.1)	18 (41.9)	
≥5,000	42 (16.7)	21 (50.0)	21 (50.0)	
Employment status^3^				0.42
Unemployed	118 (46.6)	69 (58.5)	49 (41.5)	
Non-precarious employment	45 (17.8)	28 (62.2)	17 (37.8)	
Precarious employment	78 (30.8)	48 (61.5)	30 (38.5)	
Self-employed	11 (4.3)	9 (81.8)	2 (18.2)	
Unpaid family worker	1 (0.4)	0 (0.0)	1 (100.0)	
Residential area^3^				0.95
Seoul metropolitan city	104 (40.8)	62 (59.6)	42 (40.4)	
Other metropolitan cities^4^	42 (16.5)	26 (61.9)	16 (38.1)	
Other cities and counties	109 (42.7)	67 (61.5)	42 (38.5)	
Data collection				0.33
Healthcare institutions	130 (46.8)	79 (60.8)	51 (39.2)	
Korea Queer Culture Festivals (Seoul, Daegu)	22 (7.9)	11 (50.0)	11 (50.0)	
Online/offline transgender communities	126 (45.3)	83 (65.9)	43 (34.1)	

Values are presented as number (%). KRW, Korean won.

1 Gender identity spectrum included transfeminine (transwomen and non-binary men) and transmasculine (transmen and non-binary women).

2 p-value of the chi-square test comparing prevalence of gender identity spectrum across sociodemographic groups.

3 Not all study participants provided this information; Number of non-responses: educational level (n=24), annual household income (n=26), employment status (n=25), and residential area (n=23).

4 Included Sejong metropolitan autonomous city.

**Table 3.** Distribution of study population and prevalence of transition-related healthcare by socio-demographic characteristics among transgender adults in Korea

	Transition-related healthcare								
	GID diagnosis (n=278)			Hormone therapy (n=276)			Sex reassignment surgery (n=271)		
	Respondents who were diagnosed with GID (n=253)^1^	Respondents who were not diagnosed with GID (n=25)	p-value^2^	Respondents who received hormone therapy (n=243)^3^	Respondents who did not receive hormone therapy (n=33)	p-value^4^	Respondents who underwent sex reassignment surgery (n=115)^5^	Respondents who did not undergo sex reassignment surgery (n=156)	p-value^6^
Gender identity spectrum^7^			0.005			<0.001			<0.001
Transfeminine	164 (94.8)	9 (5.2)		160 (93.6)	11 (6.4)		57 (33.9)	111 (66.1)	
Transmasculine	89 (84.8)	16 (15.2)		83 (79.0)	22 (21.0)		58 (56.3)	45 (43.7)	
Age (yr)			0.02			0.006			0.71
19-29	194 (89.0)	24 (11.0)		185 (85.3)	32 (14.7)		90 (41.9)	125 (58.1)	
30-50	59 (98.3)	1 (1.7)		58 (98.3)	1 (1.7)		25 (44.6)	31 (55.4)	
Sexual orientation			0.26			0.70			0.001
Heterosexual	120 (93.0)	9 (7.0)		116 (89.9)	13 (10.1)		68 (54.4)	57 (45.6)	
Homosexual	24 (82.8)	5 (17.2)		23 (82.1)	5 (17.9)		11 (39.3)	17 (60.7)	
Bisexual	86 (89.6)	10 (10.4)		83 (87.4)	12 (12.6)		26 (27.7)	68 (72.3)	
Asexual	23 (95.8)	1 (4.2)		21 (87.5)	3 (12.5)		10 (41.7)	14 (58.3)	
Educational level^8^			0.73			0.88			0.28
≤High school graduate	53 (93.0)	4 (7.0)		52 (91.2)	5 (8.8)		19 (33.3)	38 (66.7)	
College graduate	35 (89.7)	4 (10.3)		34 (87.2)	5 (12.8)		17 (43.6)	22 (56.4)	
University graduate	134 (92.4)	11 (7.6)		128 (88.3)	17 (11.7)		68 (46.9)	77 (53.1)	
≥Graduate school graduate	11 (84.6)	2 (15.4)		11 (84.6)	2 (15.4)		4 (30.8)	9 (69.2)	
Annual household income (10^4^ KRW)^8^			0.38			0.21			0.10
<1,000	61 (88.4)	8 (11.6)		58 (84.1)	11 (15.9)		26 (37.7)	43 (62.3)	
1,000-1,999	51 (96.2)	2 (3.8)		51 (96.2)	2 (3.8)		29 (54.7)	24 (45.3)	
2,000-2,999	42 (93.3)	3 (6.7)		41 (91.1)	4 (8.9)		17 (37.8)	28 (62.2)	
3,000-4,999	41 (95.3)	2 (4.7)		38 (88.4)	5 (11.6)		14 (32.6)	29 (67.4)	
≥5,000	37 (88.1)	5 (11.9)		35 (83.3)	7 (16.7)		22 (52.4)	20 (47.6)	
Employment status^8^			0.006			0.002			0.68
Unemployed	105 (89.0)	13 (11.0)		98 (83.1)	20 (16.9)		54 (45.8)	64 (54.2)	
Non-precarious employment	42 (93.3)	3 (6.7)		42 (93.3)	3 (6.7)		19 (42.2)	26 (57.8)	
Precarious employment	74 (94.9)	4 (5.1)		74 (94.9)	4 (5.1)		29 (37.2)	49 (62.8)	
Self-employed	11 (100.0)	0 (0.0)		11 (100.0)	0 (0.0)		4 (36.4)	7 (63.6)	
Unpaid family worker	0 (0.0)	1 (100.0)		0 (0.0)	1 (100.0)		0 (0.0)	1 (100.0)	
Residential area^8^			0.07			0.08			0.02
Seoul metropolitan city	99 (95.2)	5 (4.8)		96 (92.3)	8 (7.7)		52 (50.0)	52 (50.0)	
Other metropolitan cities^9^	40 (95.2)	2 (4.8)		39 (92.9)	3 (7.1)		21 (50.0)	21 (50.0)	
Other cities and counties	95 (87.2)	14 (12.8)		91 (83.5)	18 (16.5)		35 (32.1)	74 (67.9)	
Data collection			0.001			0.005			0.007
Healthcare institutions	127 (97.7)	3 (2.3)		123 (94.6)	7 (5.4)		42 (32.6)	87 (67.4)	
Korea Queer Culture Festivals (Seoul, Daegu)	19 (86.4)	3 (13.6)		17 (77.3)	5 (22.7)		12 (54.5)	10 (45.5)	
Online/offline transgender communities	107 (84.9)	19 (15.1)		103 (83.1)	21 (16.9)		61 (50.8)	59 (49.2)	

Values are presented as number (%). GID, gender identity disorder; KRW, Korean won.

1 Not all study participants answered each question; Number of respondents diagnosed with GID who provided: educational level (n=233), annual household income and employment status (n=232), and residential area (n=234).

2 p-value of the chi-square test comparing the prevalence of being or not being diagnosed with GID across different sociodemographic groups.

3 Not all study participants answered each question; Number of respondents who received hormone therapy who provided: educational level (n=225), annual household income (n=223), employment status (n=225), and residential area (n=226).

4 p-value of the chi-square test comparing the prevalence of receiving or not receiving hormone therapy across different sociodemographic groups.

5 Not all study participants answered each question; Number of respondents who had at least one sex reassignment surgery who provided: educational level and annual household income (n=108), employment status (n=106), and residential area (n=108).

6 p-value of the chi-square test comparing the prevalence of having or not having sex reassignment surgery across different sociodemographic groups.

7 Gender identity spectrum included transfeminine (transwomen and non-binary men) and transmasculine (transmen and non-binary women).

8 Not all study participants provided this information; Number of non-responses: educational level (n=24), annual household income (n=26), employment status (n=25), and residential area (n=23).

9 Included Sejong metropolitan autonomous city.

**Table 4.** Experience of diagnosis with GID and reasons for not pursuing GID diagnosis

GID diagnosis	Distribution (n=278)	Gender identity spectrum^1^	
		Transfeminine (n=173)	Transmasculine (n=105)
Yes	253 (91.0)	164 (94.8)	89 (84.8)
No	25 (9.0)	9 (5.2)	16 (15.2)
Reason for not pursing GID diagnosis (n=25)^2^			
I was having financial difficulties	12 (48.0)	4 (44.4)	8 (50.0)
I was debating whether I should see a doctor for a diagnosis	11 (44.0)	2 (22.2)	9 (56.3)
I am currently not in need of a diagnosis of gender identity disorder	11 (44.0)	3 (33.0)	8 (50.0)
I was worried about possible discrimination associated with a psychiatric diagnosis on my medical history	9 (36.0)	3 (33.0)	6 (37.5)
I could not find a psychiatrist who would provide an adequate diagnosis	7 (28.0)	2 (22.2)	5 (31.3)
I am currently seeing a counsellor/psychiatrist for counselling services	7 (28.0)	3 (33.3)	4 (25.0)
I was concerned with the stigma associated with having a mental disorder	4 (16.0)	3 (33.3)	1 (6.3)
My family and/or friends advised against it	1 (4.0)	0 (0.0)	1 (6.3)
I was refused a diagnosis of gender identity disorder	0 (0.0)	0 (0.0)	0 (0.0)
Other reasons	3 (12.0)	1 (11.1)	2 (12.5)

Values are presented as number (%). GID, gender identity disorder.

1 Gender identity spectrum included transfeminine (transwomen and non-binary men) and transmasculine (transmen and non-binary women).

2 Respondents could select multiple choices.

**Table 5.** Experience of hormone therapy and reasons for not currently receiving hormone therapy

Currently receiving hormone therapy	Distribution (n=276)	Gender identity spectrum^1^	
		Transfeminine (n=171)	Transmasculine (n=105)
Yes	225 (81.5)	150 (87.7)	75 (71.4)
No	51 (18.5)	21 (12.3)	30 (28.6)
Reason for not currently receiving hormone therapy (n=51)^2^			
I was having financial difficulties	28 (54.9)	10 (47.6)	18 (60.0)
I believed it would raise difficulties in my economic activities such as finding employment and working	24 (47.1)	8 (38.1)	16 (53.3)
It raised health issues	23 (45.1)	9 (42.9)	14 (46.7)
I did not have a medical certificate from a psychiatrist	17 (33.3)	4 (19.0)	13 (43.3)
I was debating whether I should receive hormone therapy	15 (29.4)	2 (9.5)	13 (43.3)
I was worried about potential stigma from other people around me	13 (25.5)	5 (23.8)	8 (26.7)
My family and/or friends advised against it	8 (15.7)	1 (4.8)	7 (23.3)
I did not have access to healthcare facilities that provided hormone therapy	8 (15.7)	3 (14.3)	5 (16.7)
I did not think hormone therapy was necessary	7 (13.7)	3 (14.3)	4 (13.3)
I was preparing for a sex reassignment surgery	4 (7.8)	3 (14.3)	1(3.3)
The sex reassignment surgery has successfully altered my physical appearance	3 (5.9)	2 (9.5)	1 (3.3)
I believed it would reduce my reproductive capacity	2 (3.9)	1 (4.8)	1 (3.3)
Other reasons	10 (19.6)	3 (14.3)	7 (23.3)

Values are presented as number (%).

1 Gender identity spectrum included transfeminine (transwomen and non-binary men) and transmasculine (transmen and non-binary women).

2 Respondents could select multiple choices.

**Table 6.** Experience of, routes to, and reasons for purchasing hormone medications without a prescription

Ever purchased hormone medications without a prescription	Distribution (n=243)	Gender identity spectrum^1^	
		Transfeminine (n=160)	Transmasculine (n=83)
No	182 (74.9)	109 (68.1)	73 (88.0)
Yes	61 (25.1)	51 (31.9)	10 (12.0)
Ever purchased hormone medications without a prescription (yes; n=61)^2^			
Over-the-counter medicines	28 (45.9)	26 (51.0)	2 (20.0)
Online foreign vendors/suppliers	19 (31.1)	18 (35.3)	1 (10.0)
Acquaintances/friends	15 (24.6)	8 (15.7)	7 (70.0)
Online domestic vendors/suppliers	10 (16.4)	9 (17.6)	1 (10.0)
Offline vendors/suppliers	5 (8.2)	4 (7.8)	1 (10.0)
Reason for ever purchasing hormone medications without a prescription (n=61)^2^			
I did not have a diagnosis from a psychiatrist	34 (55.7)	28 (54.9)	6 (60.0)
I did not know healthcare facilities where I could acquire prescription for hormone medications	15 (24.6)	11 (21.6)	4 (40.0)
I did not want to have a record of medical treatment on my medical history	10 (16.4)	8 (15.7)	2 (20.0)
Purchase hormone medications without a prescription was a cheaper choice	10 (16.4)	6 (11.8)	4 (40.0)
I did not want to go to a hospital	10 (16.4)	6 (11.8)	4 (40.0)
Other reasons	17 (27.9)	14 (27.5)	3 (30.0)

Values are presented as number (%).

1 Gender identity spectrum included transfeminine (transwomen and non-binary men) and transmasculine (transmen and non-binary women).

2 Respondents could select multiple choices.

**Table 7.** Experience of sex reassignment surgery and reasons for not having sex reassignment surgery

Sex reassignment surgery	Distribution (n=271)	Gender identity spectrum^1^	
		Transfeminine (n=168)	Transmasculine (n=103)
Yes	115 (42.4)	57 (33.9)	58 (56.3)
No	156 (57.6)	111 (66.1)	45 (43.7)
Reason for not having sex reassignment surgery (n=156)^2^			
The surgery was too costly	122 (78.2)	85 (76.6)	37 (82.2)
I wish to receive the surgery eventually, but I am not ready for it right now	100 (64.1)	68 (61.3)	32 (71.1)
I believed it would raise difficulties in my economic activities such as finding employment and working	57 (36.5)	37 (33.3)	20 (44.4)
My family and/or friends advised against it	39 (25.0)	30 (27.0)	9 (20.0)
The dangers of the surgery were too high	36 (23.1)	27 (24.3)	9 (20.0)
I was worried about potential stigma from other people around me	32 (20.5)	22 (19.8)	10 (22.2)
I have not met the medical requirements for the surgery	27 (17.3)	19 (17.1)	8 (17.8)
The surgery would not guarantee satisfactory sexual function	19 (12.2)	9 (8.1)	10 (22.2)
I did not have access to healthcare facilities that provided sex reassignment surgery	11 (7.1)	6 (5.4)	5 (11.1)
I did not think sex reassignment surgery was necessary	10 (6.4)	5 (4.5)	5 (11.1)
It raised issues regarding family planning and reproduction such as pregnancy and childbirth	7 (4.5)	3 (2.7)	4 (8.9)
I was refused a sex reassignment surgery	1 (0.6)	1 (0.9)	0 (0.0)
Other reasons	13 (8.3)	10 (9.0)	3 (6.7)

Values are presented as number (%).

1 Gender identity spectrum included transfeminine (transwomen and non-binary men) and transmasculine (transmen and non-binary women).

2 Respondents could select multiple choices.

## SUPPLEMENTARY MATERIALS

Supplementary Material: Korean version is available at http://www.e-epih.org/.

## Appendix 1. Cost^1^ of gender identity disorder (GID) diagnosis and sex reassignment surgery

**Table:**

Cost of GID diagnosis (n=251)	Distribution	Gender identity spectrum^2^			
		Transfeminine (n=162)		Transmasculine(n=89)	
	n (%)	n (%)		n (%)	
<25	42 (16.7)	26 (16.0)		16 (18.0)	
25-49	170 (67.7)	110 (67.9)		60 (67.4)	
50-74	22 (8.8)	11 (6.8)		11 (12.4)	
>74	17 (6.8)	15 (9.3)		2 (2.2)	

**Sex reassignment surgery (n=115)**	**Distribution**	**Transfeminine (n=57)**		**Transmasculine(n=58)**	
	**n**	**n**	**Average cost (SD)**	**n**	**Average cost (SD)**

Breast/chest	71	17	532.4 (443.3)	54	368.5 (146.4)
Orchiectomy, hysterectomy/salpingo-oophorectomy	63	21	297.1 (523.8)	42	397.6 (290.1)
Genital reconstruction	34	27	1,514.8 (657.3)	7	2,057.1 (1,135.6)
Face	30	27	1,159.6 (1,048.5)	3	366.7 (57.7)
Voice	9	9	655.6 (545.7)	-	-
Others	9	7	132.9 (118.1)	2	100 (0.0)

1 10,000 Korean won.

2 Gender identity spectrum included transfeminine (transwomen and non-binary men) and transmasculine (transmen and non-binary women).

## Appendix 2. Distribution of study population and experiences of delaying or avoiding hospital visits and being denied healthcare services among transgender adults in Korea within the past 12 months

**Table:**

	Distribution (n=262)	Delaying or avoiding hospital visits (n=112)		Being denied healthcare services (n=14)	
		p-value^1^		p-value^2^	
Gender identity spectrum^3^			0.02		0.17
Transfeminine	160 (61.1)	59 (36.9)		11 (6.9)	
Transmasculine	102 (38.9)	53 (52.0)		3 (2.9)	
Age (yr)			0.04		0.91
19-29	209 (79.8)	96 (45.9)		11 (5.3)	
30-50	53 (20.2)	16 (30.2)		3 (5.7)	
Sexual orientation			0.27		0.003
Heterosexual	123 (46.9)	52 (42.3)		4 (3.3)	
Homosexual	26 (9.9)	9 (34.6)		2 (7.7)	
Bisexual	90 (34.4)	37 (41.1)		3 (3.3)	
Asexual	23 (8.8)	14 (60.9)		5 (21.7)	
Educational level^4^			0.24		0.62
≤High school graduate	57 (22.5)	25 (43.9)		2 (3.5)	
College graduate	39 (15.4)	12 (30.8)		2 (5.1)	
University graduate	144 (56.9)	68 (47.2)		10 (6.9)	
≥Graduate school graduate	13 (5.1)	4 (30.8)		0 (0.0)	
Household income^4^ (10^4^ KRW)			0.38		0.67
<1,000	69 (27.5)	30 (43.5)		6 (8.7)	
1,000-1,999	52 (20.7)	28 (53.8)		3 (5.8)	
2,000-2,999	45 (17.9)	16 (35.6)		1 (2.2)	
3,000-4,999	43 (17.1)	16 (37.2)		2 (4.7)	
≥5,000	42 (16.7)	17 (40.5)		2 (4.8)	
Employment status^4^			0.67		0.93
Unemployed	117 (46.4)	53 (45.3)		7 (6.0)	
Non-precarious employment	45 (17.9)	21 (46.7)		2 (4.4)	
Precarious employment	78 (31.0)	29 (37.2)		3 (3.8)	
Self-employed	11 (4.4)	5 (45.5)		1 (9.1)	
Unpaid family worker	1 (0.4)	0 (0.0)		0 (0.0)	
Residential area^4^			0.43		0.22
Seoul metropolitan city	104 (40.9)	49 (47.1)		4 (3.8)	
Other metropolitan cities^5^	42 (16.5)	15 (35.7)		1 (2.4)	
Other cities and counties	108 (42.5)	45 (41.7)		9 (8.3)	
Data collection			0.07		0.15
Healthcare institutions	127 (48.5)	48 (37.8)		7 (5.5)	
The Korean Queer Culture Festivals (Seoul, Daegu)	22 (8.4)	14 (63.6)		3 (13.6)	
Online/offline transgender communities	113 (43.1)	50 (44.2)		4 (3.5)	

Values are presented as number (%). KRW, Korean won.

1 p-value of chi-square test comparing prevalence of delaying or avoiding hospital visits across sociodemographic groups.

2 p-value of chi-square test comparing prevalence of being denied healthcare services across sociodemographic groups.

3 Gender identity spectrum included transfeminine (transwomen and non-binary men) and transmasculine (transmen and non-binary women).

4 Not all study participants provided this information. Number of non-responses: educational level (n=9), household income (n=11), employment status (n=10), and residential area (n=8).

5 Included Sejong metropolitan autonomous city.

## References

[1] American Psychological Association (2011). Answers to your questions: about transgender people, gender identity, and gender expression. http://www.apa.org/topics/lgbt/transgender.pdf.

[2] Coleman E, Bockting W, Botzer M, Cohen-Kettenis P, DeCuypere G, Feldman J (2012). Standards of care for the health of transsexual, transgender, and gender-nonconforming people, version 7. Int J Transgend.

[3] James SE, Herman JL, Rankin S, Keisling M, Mottet L, Anafi M (2016). The report of the 2015 U.S. transgender survey. https://www.transequality.org/sites/default/files/docs/USTS-Full-Report-FINAL.PDF.

[4] Reisner SL, Poteat T, Keatley J, Cabral M, Mothopeng T, Dunham E (2016). Global health burden and needs of transgender populations: a review. Lancet.

[5] Meerwijk EL, Sevelius JM (2017). Transgender population size in the United States: a meta-regression of population-based probability samples. Am J Public Health.

[6] Korean Statistical Information Service KOSIS 100 indicators. http://kosis.kr/conts/nsportalStats/nsportalStats_0102Body.jsp?menuId=10&NUM=1014.

[7] Yi H, Lee H, Yoon JW, Park J, Kim S-S (2015). Transgender people’s access to health care in Korea. Health Soc Welf Rev.

[8] Murad MH, Elamin MB, Garcia MZ, Mullan RJ, Murad A, Erwin PJ (2010). Hormonal therapy and sex reassignment: a systematic review and meta-analysis of quality of life and psychosocial outcomes. Clin Endocrinol (Oxf).

[9] Weyers S, Elaut E, De Sutter P, Gerris J, T’sjoen G, Heylens G (2009). Long-term assessment of the physical, mental, and sexual health among transsexual women. J Sex Med.

[10] Wilson EC, Chen YH, Arayasirikul S, Wenzel C, Raymond HF (2015). Connecting the dots: examining transgender women’s utilization of transition-related medical care and associations with mental health, substance use, and HIV. J Urban Health.

[11] Son I, Lee H, Park J, Kim SS (2017). Social stigma and medical marginalization in healthcare service among transgender people in South Korea. Korean J Sociol.

[12] Choi SK (2013). The sex correction of transsexuals and its standards: focusing on ‘the Supreme Court 2011.9.2. 2009스 117’ case. Korean J Fam Law.

[13] Hong SP, Lee SH (2013). A legal appraisal of the medical intervention in the gender recognition of transgender under the international human rights and the Korean law, with focus on the recent decision of the Seoul Western District Court. Korean J Int Comp Law.

[14] Park H (2016). Transgender people and their obligation to serve in the military in South Korea.

[15] Kim YJ (2012). A study of fundamental rights that the person owns transsexualism is guaranteed. [dissertation].

[16] Lee H, Park J, Kim SS (2014). LGBTQI health research in South Korea: a systematic review. Health Soc Sci.

[17] Reisner SL, Conron KJ, Tardiff LA, Jarvi S, Gordon AR, Austin SB (2014). Monitoring the health of transgender and other gender minority populations: validity of natal sex and gender identity survey items in a US national cohort of young adults. BMC Public Health.

[18] Chang SY, Kim JH, Kim HK, Na YJ, Jung HH, Ryu MH (2014). A survey on discrimination based on sexual orientation and gender identity in South Korea. https://library.humanrights.go.kr/search/media/img/CAT000000038573?metsno=000000000936&fileid=M000000000936_FILE000001.

[19] Dewey JM, Gesbeck MM (2017). (Dys) Functional diagnosing mental health diagnosis, medicalization, and the making of transgender patients. Humanit Soc.

[20] Dowshen N, Nguyen GT, Gilbert K, Feiler A, Margo KL, Stroumsa D (2014). Improving transgender health education for future doctors. Am J Public Health.

[21] Safer JD, Pearce EN (2013). A simple curriculum content change increased medical student comfort with transgender medicine. Endocr Pract.

[22] Lombardi E (2001). Enhancing transgender health care. Am J Public Health.

[23] Rotondi NK, Bauer GR, Scanlon K, Kaay M, Travers R, Travers A (2013). Nonprescribed hormone use and self-performed surgeries: “do-it-yourself” transitions in transgender communities in Ontario, Canada. Am J Public Health.

