# Supplementary material for: Experiences of and barriers to transition-related healthcare among Korean transgender adults: focus on gender identity disorder diagnosis, hormone therapy, and sex reassignment surgery
Source: Epidemiol Health. 2018 Feb 27;40:e2018005. doi: 10.4178/epih.e2018005 (PMC6060334; doi:10.4178/epih.e2018005)
Supplement: Supplementary file 1 [file epih-40-e2018005-supplementary.pdf]

Open Access

Volume: 40, Article ID: e2018005
https://doi.org/10.4178/epih.e2018005

epiH
Epidemiology and Health

ORIGINAL ARTICLE

한국 트랜스젠더의 의료적 트랜지션 관련 경험과 장벽: 정신과진단, 호르몬요법, 성전환수술을 중심으로

이혜민[1], 박주영[1], 최보경[1], 이호림[1], 김승섭[1,2]

[1]고려대학교 일반대학원 보건과학과; [2]하버드대학교 보건대학원 사회행동학과

Experiences of and barriers to transition-related healthcare among Korean transgender adults: focus on gender identity disorder diagnosis, hormone therapy, and sex reassignment surgery

Hyemin Lee[1], Jooyoung Park[1], Bokyoung Choi[1], Horim Yi[1], Seung-Sup Kim[1,2]

[1]Department of Public Health Sciences, Graduate School of Korea University, Seoul, Korea; [2]Department of Social and Behavioral Sciences, Harvard T. H. Chan School of Public Health, Boston, MA, USA

OBJECTIVES: Transgender people may encounter barriers to transition-related healthcare services. This study aimed to investigate the experiences of transition-related healthcare and barriers to those procedures among transgender adults in Korea.
METHODS: In 2017, we conducted a nationwide cross-sectional survey of 278 transgender adults, which named Rainbow Connection Project II, in Korea. We assessed the prevalence of transition-related healthcare, including gender identity disorder (GID) diagnosis, hormone therapy, and sex reassignment surgery. To understand the barriers to those procedures, we also asked participants for their reasons for not receiving each procedure. Further, this study examined their experiences of and the reasons for using non-prescribed hormone medications.
RESULTS: Of transgender people participated in the survey, 91.0% (n = 253/278) were diagnosed with GID, 88.0% (n = 243/276) received hormone therapy, and 42.4% (n = 115/271) have had any kind of sex reassignment surgery. Cost was the most common barrier to transition-related healthcare among Korean transgender adults. Other common barriers were identified as follows: negative experiences in healthcare settings, lack of specialized healthcare professionals and facilities, and social stigma against transgender people. Among those who had taken hormone medications, 25.1% (n = 61/243) reported that they had ever purchased them without a prescription.
CONCLUSIONS: Our findings suggest that barriers to transition-related healthcare exist in Korea and constrain transgender individuals' safe access to the needed healthcare. Institutional interventions are strongly recommended to improve access to transition-related healthcare. These interventions include provision of programs to train Korean healthcare professionals and expansion of national health insurance to include these procedures.

KEY WORDS: Transgender persons, Health services for transgender persons, Sex reassignment surgery, Sexual and gender minorities, Gender identity, Republic of Korea

Correspondence: Seung-Sup Kim
Department of Public Health Sciences, Graduate School of Korea University, 145 Anam-ro, Seongbuk-gu, Seoul 02841, Korea
E-mail: ssk3@korea.ac.kr
Received: Nov 29, 2017 / Accepted: Feb 27, 2018 / Published: Feb 27, 2018
This article is available from: http://e-epih.org/
This is an open-access article distributed under the terms of the Creative Commons Attribution License (http://creativecommons.org/licenses/by/4.0/), which permits unrestricted use, distribution, and reproduction in any medium, provided the original work is properly cited.
© 2017, Korean Society of Epidemiology

서 론

연구의 배경

트랜스젠더는 본인의 성별정체성(gender identity)이나 성별표현(gender expression)이 출생 시 법적성별(legal sex at birth)과 일치하지 않아 성별위화감(gender dysphoria)을 가지고 있는 사람을 가리키는 포괄적인 용어다[1,2]. 트랜스젠더는 트랜스여성(transwoman)과 트랜스남성(transman), 논바이너리 트랜스젠더

(non-binary transgender person) 등으로 분류할 수 있다. 논바이너리 트랜스젠더는 본인의 성별정체성을 남성 또는 여성과 같이 이분법적으로 규정하지 않는 이들을 말한다[3]. 트랜스젠더와 달리, 시스젠더(cisgender people)는 출생 시 법적성별과 본인의 성별정체성이 일치하는 사람을 가리킨다[4].

2017년 미국에서 트랜스젠더 인구규모를 추산한 연구에 따르면, 트랜스젠더는 인구 10만 명당 390명이라고 한다[5]. 이는 3개의 데이터베이스를 활용하여 12개의 국가단위 설문조사를 메타분석한 것으로, 기존 연구가 트랜스젠더의 인구규모를 추정하는 과정에서 가지는 비확률 표집 및 대표성 등의 문제점을 보완한 결과이다. 메타분석 결과 제시된 인구 10만 명 당 390명을 2018년 2월 기준 한국 전체 인구 51,635,256명[6]에 대입하면 국내 트랜스젠더의 인구규모는 20만 1,377명으로 추산할 수 있다.

트랜스젠더는 성별위화감을 해소하고 본인의 성별정체성에 부합하는 성별로 살아가기 위해 성별 트랜지션(gender transition)이라는 전환과정을 거친다[7]. 그 과정에서 필요할 경우 트랜스젠더는 성별위화감에 대한 정신과진단 및 호르몬요법, 성전환수술 등과 같은 의료적 조치를 받기도 하며, 이를 트랜지션 관련 의료적 조치(transition-related healthcare) 또는 의료적 트랜지션(medical transition)이라고 한다[7]. 기존 연구에 따르면, 의료적 트랜지션을 통해 트랜스젠더의 성별위화감을 완화할 수 있을 뿐만 아니라 전반적인 삶의 질과 자살생각, 성적기능 등 신체적·정신적 건강을 향상할 수 있다[8-10]. 모든 트랜스젠더가 호르몬요법이나 성전환수술을 받는 것은 아니지만[7,11], 대다수 트랜스젠더에게 이러한 의학적 조치는 건강과 삶의 질을 결정짓는 중요한 요소다 [4].

한국에서 트랜스젠더는 개인의 성별위화감을 완화하기 위한 목적뿐 아니라 법적 성별정정 또는 병역 면제와 같은 제도적 요인 때문에 의료적 트랜지션을 한다[7,11]. 트랜스젠더의 법적 성별정정은 대법원 가족관계등록예규 제435호, 「성전환자의 성별정정허가신청사건 등 사무처리지침」을 기반으로 진행된다[12,13]. 해당 예규는 법적 성별정정을 위한 조사사항과 필수 첨부서류를 통해 신청인의 정신과진단과 생식능력 제거 및 성전환수술을 요구한다. 특히, 성전환수술과 관련하여 신청인은 '성전환수술을 받아 현재 생물학적인 성과 반대되는 성에 관한 신체의 성기와 흡사한 외관을 구비하고 있음을 확인하는 성전환시술 의사의 소견서'를 첨부서류로 제출해야 한다.

또한 한국에서 법적 성별이 남성이고 한국 국적을 가지고 있는 이들은 모두 헌법 제39조제1항과 병역법에 따라 병역의 의무를 가지는데, 법적 성별을 정정하지 않은 트랜스여성에게도 동일한 의무가 부과된다[14,15]. 이러한 상황에서 병역을 면제받으려는 트랜스여성은 성주체성장애 진단, 호르몬요법과 성전환수술까지 받아야 한다[11,14,15]. 트랜스남성 또한, 남성으로 법적 성별을 정정한 이후에는 병역 의무의 대상이 되어 징병검사를 받는다[14].

그러나, 한국에서 이와 관련한 보건의료체계 및 제도는 미비하다[7,11]. 첫째, 국내 의과대학 정규 교육과정에 의료적 트랜지션에 대한 내용이 포함되어 있지 않다. 정부나 학회 차원에서도 트랜지션에 대한 의학적 지식을 전달하는 수련매뉴얼이나 가이드라인이 부재한 상황이다. 둘째, 호르몬요법과 성전환수술 등 의료적 트랜지션은 건강보험으로 보장되지 않아 트랜스젠더 개인이 그 비용을 전액 부담하고 있다.

트랜스젠더의 의료적 트랜지션 이용과 그 과정에서 경험하는 장벽에 대한 학문적 관심 또한 매우 부족하다. 2013년까지 출판된 한국 성소수자 건강 관련 논문 128편 중 22편만이 트랜스젠더를 대상으로 하였고, 22편 중 대부분은 트랜스젠더 환자의 사례보고 또는 임상 수술법을 소개하는 임상적 연구였다[16]. 병원을 기반으로 수행된 임상적 연구 외에 트랜스젠더의 의료적 트랜지션과 의료이용 경험에 대한 연구는 2017년 출판된 질적 연구 단 한 편에 불과하다[11].

연구의 목적

본 연구는 한국 성인 트랜스젠더가 참여한 단면조사 자료를 분석하여, 트랜스젠더 참여자의 정신과진단, 호르몬조치 및 성전환수술 등 의료적 트랜지션 관련 경험과 그 과정에서 마주하는 장벽을 파악하고자 하였다. 더불어, 본 연구팀은 트랜스젠더 참여자의 안전하지 않은 의료적 트랜지션 실태를 알아보기 위해, 병원의 처방없이 호르몬을 구매한 경험과 그 이유를 밝히고자 했다.

연구방법

분석대상과 자료

본 연구는 한국 트랜스젠더의 의료적 트랜지션 관련 경험과 그 과정에서의 장벽을 파악하기 위해 국내 거주 중인 성인 트랜스젠더를 대상으로 설문조사를 수행하였다. 연구참여자는 만 19세 이상 한국 성인이며, 1) 트랜스여성(출생 시 법적성별 남성), 2) 트랜스남성(출생 시 법적성별 여성), 또는 3) 호르몬조치, 성전환수술 등 의료적 트랜지션의 경험을 가지고 있는 논바이너리 트랜스젠더다. 논바이너리 트랜스젠더도 성별위화감을 완화하고자 하는 개인의 욕구뿐만 아니라 법적 성별정정 및 병역 문제 등 사회제도적 요인으로 인해 의료적 트랜지션을 받는 경우가 있기 때문에[11] 본 연구의 참여자로 포함하였다.

설문조사는 2017년 6월 27일부터 2017년 8월 31일까지 약 2개월 동안 진행되었다. 설문조사에 대한 홍보와 연구참여자 모집은 서울과 대구에서 개최된 퀴어문화축제와 다수의 트랜스젠더가 이용하는 서울 소재 의료기관 4곳, 온·오프라인 트랜스젠더 커뮤니티 3곳을 활용하였다. 설문조사는 연구 참여 및 연구의 학술적 활용에 대한 동의를 받은 이후에 진행하였다. 응답자에게는 연구

참여에 대한 보상으로 10,000원 상당의 보상을 지급했다. 본 연구는 고려대학교 기관연구윤리심의위원회의 승인(승인번호: 1040548-KU-IRB-17-67-A-1)을 받았다.

본 설문조사에는 총 347명의 트랜스젠더가 참여하였다. 그 중 연구 참여와 연구의 학술적 활용에 동의하지 않은 5명, 연령 및 성별정체성 등 인구학적 정보에 응답하지 않은 62명, 의료적 트랜지션 중 정신과진단 여부를 묻는 문항에 응답하지 않은 2명을 제외하였다. 그 결과, 트랜스젠더 278명의 자료를 본 연구의 최종 분석 대상으로 삼았다.

측정도구

설문지는 인구사회학적 정보, 성별정체성 정보, 의료적 트랜지션의 경험, 의료접근성, 법적 성별정정, 건강상태 등 총 7가지 범주로 구성되어 있다. 참여자들은 개인의 성별정체성과 의료적 트랜지션의 경험에 따른 추가 질문을 포함하여 약 160-230개 사이의 문항에 응답하였다.

연구참여자의 성별정체성은 출생 시 법적성별과 현재 성별정체성을 묻는 두 가지 문항을 활용하여, 출생 시 법적성별과 현재 성별정체성이 일치하지 않는 경우를 트랜스젠더로, 일치하는 경우를 비트랜스젠더인 시스젠더로 구분하였다(표 1)[17]. 트랜스젠더 중 출생 시 법적 성별은 남성이지만 현재 성별정체성이 여성인 이들을 트랜스여성, 반대의 경우를 트랜스남성이라고 한다. 현재 성별정체성을 남성 또는 여성으로 확고히 정체화하고 있지 않은 응답자는 논바이너리 트랜스젠더로 분류하였으며, 이들을 출생 시 법적성별이 남성인지 여성인지에 따라 구분하였다. 본 연구에서는 출생 시 법적성별이 남성인 트랜스여성과 논바이너리 트랜스젠더를 트랜스여성 범주(trans feminine spectrum)로, 출생 시 법적성별이 여성인 트랜스남성과 논바이너리 트랜스젠더를 트랜스남성 범주(trans masculine spectrum)로 분류하였다[4].

그 외 인구학적 변수로 연령, 성적지향, 거주지역을 제시하였다. 연령의 경우 만 19세-29세, 30세-50세의 두 집단으로 분류하였다. 성적지향은 동성애, 양성애, 이성애, 무성애로 구분하였다. 연구참여자의 거주지역은 서울특별시와 광역시, 시·군 지역으로 나누었다. 사회경제적 변수로는 교육수준, 연평균 가구소득, 고용형태를 포함하였다. 교육수준은 고등학교 졸업, 2년제 대학 재학·중퇴·졸업, 4년제 대학 재학·중퇴·졸업, 대학원 졸업 이상으로 분류하였다. 연평균 가구소득의 경우 1,000만원 미만, 1,000만원-1,999만원, 2,000만원-2,999만원, 3,000만원-4,999만원, 5,000만원 이상으로 분류하였다. 고용형태는 실업 또는 무직, 정규직, 비정규직, 자영업, 무급가족종사로 나누었다. 추가로, 설문조사 참여경로를 의료기관 4곳과 퀴어문화축제 2곳, 온·오프라인 커뮤니티 3곳으로 구분하여 제시하였다.

표 1. 성별정체성을 측정하는 2단계 방법[1]

현재 성별정체성	출생 시 법적 성별	
	남성	여성
남성	시스젠더[2]	트랜스남성 범주
여성	트랜스여성 범주	시스젠더[2]
남성 또는 여성으로 정체화하지 않음	트랜스여성 범주	트랜스남성 범주

[1]Reisner et al. Lancet 2016;388:412-436 [4]에 제시된 표의 내용을 재구성했다.
[2]시스젠더는 출생 시 법적 성별이 현재 성별정체성과 일치하는 비트랜스젠더를 말한다.

본 연구에서 의료적 트랜지션은 성주체성장애에 대한 정신과진단과 호르몬조치, 성전환수술로 나누어 측정하였다. 성주체성장애 진단은 "귀하는 '성주체성장애'에 대한 한국표준질병·사인분류(KCD)에 따른 정신과진단(F64.0. 성전환증 또는 F64.9. 상세 불명의 성주체성장애 등)을 받았습니까?"라는 문항에 "예" 또는 "아니오"로 응답하도록 했다. 정신과진단을 받지 않은 경우에는 추가로 그 이유에 대해 중복응답으로 답변하도록 했다.

호르몬조치는 "귀하는 현재 호르몬조치를 하고 있습니까?"라는 질문으로 측정하였고, "하고 있다", "과거에 했지만 지금 하지 않고 있다", "한 적 없지만 언젠가 하고 싶다(계획하고 있다)", "하고 싶은지 잘 모르겠다", "원하지 않는다"는 보기에 응답하도록 했다. "하고 있다"와 "과거에 했지만 지금 하지 않고 있다"에 응답한 참여자는 호르몬요법을 한 번이라도 경험한 것으로 분류하였고, 이 경우 병원의 처방없이 호르몬을 구매한 경험이 있는지 질문하였다. 병원의 처방없이 호르몬을 구매한 경험에 대해서는 구입 경로와 그 이유를 함께 물었으며, 중복응답이 가능하도록 했다. 또한, 현재 호르몬투여를 하지 않는 참여자들을 대상으로 그 이유를 파악하고자 했고, 이 역시 중복응답이 가능했다.

성전환수술의 경우, 성전환 관련 외과적 수술을 한 가지 이상 받은 경험이 있는지를 묻고, 받지 않았다면 그 이유를 함께 측정하였다. 성전환수술을 받지 않은 이유는 중복응답으로 답변하도록 했다.

본 연구는 추가로 성별정체성에 따른 정신과진단 및 성전환수술에 드는 비용을 측정하였다. 정신과진단 비용은 참여자가 0만원부터 100만원 사이에서 선택하도록 했다. 호르몬요법은 투여 기간에 따라 소요되는 총 비용이 달라지기 때문에 평균을 산출하여 제시하지 않았다. 성전환수술은 가슴수술, 고환·정소/난소·자궁 제거수술, 성기성형수술, 안면성형수술, 성대(목소리)수술(출생 시 법적성별 남성만), 기타수술로 분류하였다. 성전환수술 비용은 0만원부터 5,000만원 사이에서 응답하도록 했으며, 평균 수술비용과 표준편차를 제시하였다(부록 표 1).

마지막으로, 트랜스젠더 참여자에게 일반적인 건강문제나 의료적 트랜지션을 위해 의료이용 시 의료기관 방문을 회피하거나 연기한 경험과 의료인으로부터 진료를 거부당한 경험에 대해 질문하였다(부록 표 2). 지난 12개월 동안 "의료기관 방문을 회피하거

나 연기한 적이 있다", "의료인으로부터 진료를 거부당한 적이 있다"는 문항에 참여자들이 각각 "있다" 또는 "없다"로 응답하도록 했다.

분석방법

본 연구는 한국 성인 트랜스젠더의 의료적 트랜지션 경험과 그 과정에서 겪은 장벽을 분석하여 기술통계로 제시하였다. 성별정체성에 따라 출생 시 법적성별이 남성인 트랜스여성과 논바이너리 트랜스젠더를 트랜스여성 범주로, 출생 시 법적성별이 여성인 트랜스남성과 논바이너리 트랜스젠더를 트랜스남성 범주로 분류하여 분석하였다. 모든 통계분석은 STATA/SE version 13.0을 이용하였다.

결 과

인구사회학적 특성에 따른 의료적 트랜지션 경험

한국 성인 트랜스젠더 건강 연구에 참여한 278명의 인구사회학적 특성에 따른 의료적 트랜지션 분포를 살펴보았다(표 2). 연구참

표 2. 한국 트랜스젠더의 인구사회학적 특성에 따른 성별정체성 범주 분포

	전체 응답자 (n=278)	성별정체성 범주[1]		p-value[2]
		트랜스여성 범주(n=173)	트랜스남성 범주(n=105)	
	n (%)	n (%)	n (%)	
연령(세)				<0.001
19-29	218 (78.4)	124 (56.9)	94 (43.1)	
30-50	60 (21.6)	49 (81.7)	11 (18.3)	
성적지향				0.001
이성애	129 (46.4)	65 (50.4)	64 (49.6)	
동성애	29 (10.4)	24 (82.8)	5 (17.2)	
양성애	96 (34.5)	66 (68.8)	30 (31.3)	
무성애	24 (8.6)	18 (75.0)	6 (25.0)	
교육 수준[3]				0.866
고등학교 졸업	57 (22.4)	34 (59.7)	23 (40.4)	
2년제 대학 재학·중퇴·졸업	39 (15.4)	25 (64.1)	14 (35.9)	
4년제 대학 재학·중퇴·졸업	145 (57.1)	86 (59.3)	59 (40.7)	
대학원 이상	13 (5.1)	9 (69.2)	4 (30.8)	
연평균 가구소득[3]				0.244
1,000만원 미만	69 (27.4)	48 (69.6)	21 (30.4)	
1,000만원-1,999만원	53 (21.0)	30 (56.6)	23 (43.4)	
2,000만원-2,999만원	45 (17.9)	30 (66.7)	15 (33.3)	
3,000만원-4,999만원	43 (17.1)	25 (58.1)	18 (41.9)	
5,000만원 이상	42 (16.7)	21 (50.0)	21 (50.0)	
고용 형태[3]				0.418
실업 또는 무직	118 (46.6)	69 (58.5)	49 (41.5)	
정규직	45 (17.8)	28 (62.2)	17 (37.8)	
비정규직	78 (30.8)	48 (61.5)	30 (38.5)	
자영업	11 (4.3)	9 (81.8)	2 (18.2)	
무급가족종사자	1 (0.4)	0 (0.0)	1 (100.0)	
거주 지역[3]				0.950
서울특별시	104 (40.8)	62 (59.6)	42 (40.4)	
광역시[4]	42 (16.5)	26 (61.9)	16 (38.1)	
시·군 지역	109 (42.7)	67 (61.5)	42 (38.5)	
설문조사 참여 경로				0.328
의료 기관	130 (46.8)	79 (60.8)	51 (39.2)	
퀴어문화축제(서울, 대구)	22 (7.9)	11 (50.0)	11 (50.0)	
온라인·오프라인 트랜스젠더 커뮤니티	126 (45.3)	83 (65.9)	43 (34.1)	

[1]성별정체성 범주는 출생 시 법적 성별이 남성인 트랜스여성 범주(트랜스여성와 논바이너리 남성)와 출생 시 법적 성별이 여성인 트랜스남성 범주(트랜스남성과 논바이너리 여성)을 포함한다.
[2]인구사회학적 변수에 따른 성별정체성 범주의 빈도를 비교한 카이제곱 검정의 p-value.
[3]각 항목별 무응답자 수는 다음과 같다: 교육 수준(n=24), 연평균 가구소득(n=26), 고용 형태(n=25), 거주지역 (n=23).
[4]세종특별자치시를 포함한다.

표 3. 한국 트랜스젠더의 인구사회학적 특성에 따른 의료적 트랜지션 이용률

	의료적 트랜지션 이용률								
	성주체성장애 진단(n=278)			호르몬요법(n=276)			성전환수술(n=271)		
	정신과진단을 받은 응답자 (n=253)[1]	정신과진단을 받지 않은 응답자(n=25)	p-value[2]	호르몬요법을 받은 응답자 (n=243)[3]	호르몬요법을 받지 않은 응답자(n=33)	p-value[4]	성전환수술을 받은 응답자 (n=115)[5]	성전환수술을 받지 않은 응답자(n=156)	p-value[6]
	n (%)	n (%)		n (%)	n (%)		n (%)	n (%)	
성별정체성 범주[7]			0.005			<0.001			<0.001
트랜스여성 범주	164 (94.8)	9 (5.2)		160 (93.6)	11 (6.4)		57 (33.9)	111 (66.1)	
트랜스남성 범주	89 (84.8)	16 (15.2)		83 (79.0)	22 (21.0)		58 (56.3)	45 (43.7)	
연령(세)			0.025			0.006			0.707
19-29	194 (89.0)	24 (11.0)		185 (85.3)	32 (14.7)		90 (41.9)	125 (58.1)	
30-50	59 (98.3)	1 (1.7)		58 (98.3)	1 (1.7)		25 (44.6)	31 (55.4)	
성적지향			0.264			0.704			0.001
이성애	120 (93.0)	9 (7.0)		116 (89.9)	13 (10.1)		68 (54.4)	57 (45.6)	
동성애	24 (82.8)	5 (17.2)		23 (82.1)	5 (17.9)		11 (39.3)	17 (60.7)	
양성애	86 (89.6)	10 (10.4)		83 (87.4)	12 (12.6)		26 (27.7)	68 (72.3)	
무성애	23 (95.8)	1 (4.2)		21 (87.5)	3 (12.5)		10 (41.7)	14 (58.3)	
교육 수준[8]			0.734			0.876			0.277
고등학교 졸업	53 (93.0)	4 (7.0)		52 (91.2)	5 (8.8)		19 (33.3)	38 (66.7)	
2년제 대학 재학·중퇴·졸업	35 (89.7)	4 (10.3)		34 (87.2)	5 (12.8)		17 (43.6)	22 (56.4)	
4년제 대학 재학·중퇴·졸업	134 (92.4)	11 (7.6)		128 (88.3)	17 (11.7)		68 (46.9)	77 (53.1)	
대학원 이상	11 (84.6)	2 (15.4)		11 (84.6)	2 (15.4)		4 (30.8)	9 (69.2)	
연평균 가구소득[8]			0.385			0.211			0.104
1,000만원 미만	61 (88.4)	8 (11.6)		58 (84.1)	11 (15.9)		26 (37.7)	43 (62.3)	
1,000만원-1,999만원	51 (96.2)	2 (3.8)		51 (96.2)	2 (3.8)		29 (54.7)	24 (45.3)	
2,000만원-2,999만원	42 (93.3)	3 (6.7)		41 (91.1)	4 (8.9)		17 (37.8)	28 (62.2)	
3,000만원-4,999만원	41 (95.3)	2 (4.7)		38 (88.4)	5 (11.6)		14 (32.6)	29 (67.4)	
5,000만원 이상	37 (88.1)	5 (11.9)		35 (83.3)	7 (16.7)		22 (52.4)	20 (47.6)	
고용 형태[8]			0.006			0.002			0.681
실업 또는 무직	105 (89.0)	13 (11.0)		98 (83.1)	20 (16.9)		54 (45.8)	64 (54.2)	
정규직	42 (93.3)	3 (6.7)		42 (93.3)	3 (6.7)		19 (42.2)	26 (57.8)	
비정규직	74 (94.9)	4 (5.1)		74 (94.9)	4 (5.1)		29 (37.2)	49 (62.8)	
자영업	11 (100.0)	0 (0.0)		11 (100.0)	0 (0.0)		4 (36.4)	7 (63.6)	
무급가족종사자	0 (0.0)	1 (100.0)		0 (0.0)	1 (100.0)		0 (0.0)	1 (100.0)	
거주 지역[8]			0.069			0.082			0.017
서울특별시	99 (95.2)	5 (4.8)		96 (92.3)	8 (7.7)		52 (50.0)	52 (50.0)	
광역시[9]	40 (95.2)	2 (4.8)		39 (92.9)	3 (7.1)		21 (50.0)	21 (50.0)	
시·군 지역	95 (87.2)	14 (12.8)		91 (83.5)	18 (16.5)		35 (32.1)	74 (67.9)	
설문조사 참여 경로			0.001			0.005			0.007
의료 기관	127 (97.7)	3 (2.3)		123 (94.6)	7 (5.4)		42 (32.6)	87 (67.4)	
퀴어문화축제(서울, 대구)	19 (86.4)	3 (13.6)		17 (77.3)	5 (22.7)		12 (54.5)	10 (45.5)	
온라인·오프라인 트랜스젠더 커뮤니티	107 (84.9)	19 (15.1)		103 (83.1)	21 (16.9)		61 (50.8)	59 (49.2)	

[1]진단을 받은 응답자 중 각 변수의 응답자 수는 다음과 같다: 교육 수준(n=233), 연평균 가구소득과 고용 형태(n=232), 거주 지역(n=234).
[2]인구사회학적 변수에 따른 성주체성장애 진단 경험 빈도를 비교한 카이제곱 검정의 p-value.
[3]호르몬요법을 받은 응답자 중 각 변수의 응답자 수는 다음과 같다: 교육 수준(n=225), 연평균 가구소득(n=223), 고용 형태(n=225), 거주 지역(n=226).
[4]인구사회학적 변수에 따른 호르몬요법 경험 빈도를 비교한 카이제곱 검정의 p-value.
[5]한 가지 종류 이상의 성전환수술을 받은 응답자 중 각 변수의 응답자 수는 다음과 같다: 교육 수준과 연평균 가구소득(n=108), 고용 형태(n=106), 거주 지역(n=108).
[6]인구사회학적 변수에 따른 성전환수술 경험 빈도를 비교한 카이제곱 검정의 p-value.
[7]성별정체성 범주는 출생 시 법적 성별이 남성인 트랜스여성 범주(트랜스여성와 논바이너리 남성)와 출생 시 법적 성별이 여성인 트랜스남성 범주(트랜스남성과 논바이너리 여성)을 포함한다.
[8]각 항목별 무응답자 수는 다음과 같다: 교육 수준(n=24), 연평균 가구소득(n=26), 고용 형태(n=25), 거주 지역(n=23).
[9]세종특별자치시를 포함한다.

여자를 성별정체성으로 나누어 보았을 때, 전체 278명 중 트랜스여성 범주는 173명(62.2%)이었으며, 트랜스남성 범주는 105명(37.8%)이었다. 전체 연구참여자 중 19-29세는 218명(78.4%), 30-50세는 60명(21.6%)으로, 20대 응답자가 약 80%를 차지했다. 성적지향은 이성애자(n=129, 46.4%)가 가장 많았으며, 그 다음으로 양성애자(n=96, 34.5%), 동성애자(n=29, 10.4%), 무성애자(n=24, 8.6%) 순이었다. 교육수준은 4년제 대학교 재학·중퇴·졸업이 145명(57.1%)으로 가장 높은 비중을 차지했으며, 고등학교 졸업 이하(n=57, 22.4%), 2년제 대학 재학·중퇴·졸업(n=39, 15.4%), 대학원 이상(n=13, 5.1%) 순으로 나타났다. 연평균 가구소득은 1,000만원 미만(n=69, 27.4%)과 1,000만원-1,999만원(n=53, 21.0%)이 절반 가까이 차지하였다. 고용형태의 경우, 실업 또는 무직(n=118, 46.6%)과 비정규직(n=78, 30.8%)이 가장 많은 것으로 나타났다. 참여자의 대다수는 시·군 지역(n=109, 42.7%)과 서울특별시(n=104, 40.8%)에 거주하고 있었다. 참여자들은 의료기관(n=130, 46.8%)을 통해 설문조사에 가장 많이 참여하였으며, 그 다음으로 온·오프라인 성소수자 커뮤니티(n=126, 45.3%), 서울 및 대구 퀴어문화축제(n=22, 7.9%) 순이었다.

의료적 트랜지션은 성주체성장애에 대한 정신과진단과 호르몬요법, 성전환수술로 구분하여 제시하였다(표 3). 총 278명의 트랜스젠더 중 성주체성장애 진단을 받은 참여자는 253명(91.0%), 호르몬요법을 현재 받고 있거나 과거에 받은 경험이 있는 참여자는 276명 중 243명(88.0%), 한 가지 이상 성전환수술을 받은 참여자는 271명 중 115명(42.4%)이었다.

성주체성장애 진단(n=278)을 받은 비율은 트랜스남성 범주(n=89, 84.8%)에 비해 트랜스여성 범주(n=164, 94.8%)에서, 20대(n=194, 89.0%)보다 30대 이상(n=59, 98.3%)에서 더 높은 것으로 보고되었다. 호르몬요법(n=276) 또한 트랜스남성 범주(n=83, 79.0%)에 비해 트랜스여성 범주(n=160, 93.6%)에서, 20대(n=185, 85.3%)보다는 30대 이상(n=58, 98.3%)에서 그 비율이 더 높았다. 성전환수술(n=271)은 트랜스여성 범주(n=57, 33.9%)보다는 트랜스남성 범주(n=58, 56.3%)에서 더 많이 받는 것으로 파악할 수 있었다.

의료적 트랜지션 관련 장벽

트랜스여성 범주 173명 중 164명(94.8%), 트랜스남성 범주의 경우 105명 중 89명(84.8%)이 성주체성장애에 대한 정신과진단을 받았다고 보고하였다(표 4). 성주체성장애 진단을 받지 않은 25명(9.0%)에게 그 이유에 대해 질문한 결과, 12명의 참여자(48.0%)가 "경제적 부담 때문에"라고 응답하였다. 다른 이유로는 "아직 고민 중이라서"(n=11, 44.0%), "현재 당장 진단서가 필요하지 않아서"(n=11, 44.0%) 등이 제시되었다.

연구에 참여한 트랜스젠더 276명 중 225명(81.5%)이 현재 호르몬요법을 받고 있다고 응답하였다(표 5). 이를 성별정체성별로 구분하면, 트랜스여성 범주 171명 중 150명(87.7%), 트랜스남성 범주 105명 중 75명(71.4%)이 호르몬요법을 받고 있었다. 현재 호르몬조치를 받고 있지 않은 51명(18.5%)을 대상으로 호르몬조치를 하지 않거나 중단한 이유를 측정하였다. 참여자들은 "경제적 부담 때문에"(n=28, 54.9%)를 가장 많이 꼽았고, 그 다음으로는 "구직, 직장생활 등 경제적 활동에 어려움이 생길 것 같아서"(n=24,

표 4. 한국 트랜스젠더의 성주체성장애 진단 여부와 진단받지 않은 이유

성주체성장애 진단 여부	분포 (n=278)	성별정체성 범주[1]	
		트랜스여성 범주(n=173)	트랜스남성 범주(n=105)
	n (%)	n (%)	n (%)
받았다	253 (91.0)	164 (94.8)	89 (84.8)
받지 않았다	25 (9.0)	9 (5.2)	16 (15.2)
성주체성장애 진단을 받지 않은 이유(n=25)[2]			
경제적 부담 때문에	12 (48.0)	4 (44.4)	8 (50.0)
아직 고민 중이라서	11 (44.0)	2 (22.2)	9 (56.3)
현재 당장 진단서가 필요하지 않아서	11 (44.0)	3 (33.0)	8 (50.0)
정신과 진단이 의료기록에 남아서 불이익을 받을까봐	9 (36.0)	3 (33.0)	6 (37.5)
제대로 진단해 주는 정신과를 찾을 수 없어서	7 (28.0)	2 (22.2)	5 (31.3)
현재 심리 상담 혹은 정신과 상담을 받고 있는 중이라서	7 (28.0)	3 (33.3)	4 (25.0)
정신장애로 취급받는 것이 싫어서	4 (16.0)	3 (33.3)	1 (6.3)
가족, 지인의 반대로	1 (4.0)	0 (0.0)	1 (6.3)
진단서 발급을 거부당함	0 (0.0)	0 (0.0)	0 (0.0)
기타	3 (12.0)	1 (11.1)	2 (12.5)

[1]성별정체성 범주는 출생 시 법적 성별이 남성인 트랜스여성 범주(트랜스여성와 논바이너리 남성)와 출생 시 법적 성별이 여성인 트랜스남성 범주(트랜스남성과 논바이너리 여성)을 포함한다.
[2]성주체성장애 진단을 받지 않은 이유는 중복 선택이 가능하다.

표 5. 한국 트랜스젠더의 호르몬요법 여부와 투여 받지 않은 이유

현재 호르몬요법 여부	분포 (n=276)	성별정체성 범주[1]	
		트랜스여성 범주(n=171)	트랜스남성 범주(n=105)
	n (%)	n (%)	n (%)
받고 있다	225 (81.5)	150 (87.7)	75 (71.4)
받고 있지 않다	51 (18.5)	21 (12.3)	30 (28.6)
현재 호르몬요법을 받고 있지 않은 이유(n=51)[2]			
경제적 부담 때문에	28 (54.9)	10 (47.6)	18 (60.0)
구직, 직장생활 등 경제적 활동에 어려움이 생길 것 같아서	24 (47.1)	8 (38.1)	16 (53.3)
건강상의 이유 때문에	23 (45.1)	9 (42.9)	14 (46.7)
정신과 진단이 없어서	17 (33.3)	4 (19.0)	13 (43.3)
아직 고민 중이라서	15 (29.4)	2 (9.5)	13 (43.3)
주변 사람들의 눈이 두려워서	13 (25.5)	5 (23.8)	8 (26.7)
가족, 지인의 반대로	8 (15.7)	1 (4.8)	7 (23.3)
갈 수 있는 의료 기관이 없어서	8 (15.7)	3 (14.3)	5 (16.7)
호르몬요법이 필요하지 않다고 생각해서	7 (13.7)	3 (14.3)	4 (13.3)
성전환수술을 준비하면서	4 (7.8)	3 (14.3)	1(3.3)
수술로 외형적인 외모 변화가 다 되어서	3 (5.9)	2 (9.5)	1 (3.3)
생식능력이 저하되기 때문에	2 (3.9)	1 (4.8)	1 (3.3)
기타	10 (19.6)	3 (14.3)	7 (23.3)

[1]성별정체성 범주는 출생 시 법적 성별이 남성인 트랜스여성 범주(트랜스여성와 논바이너리 남성)와 출생 시 법적 성별이 여성인 트랜스남성 범주(트랜스남성과 논바이너리 여성)을 포함한다.
[2]현재 호르몬요법을 받고 있지 않은 이유는 중복 선택이 가능하다.

표 6. 한국 트랜스젠더의 병원 처방 없는 호르몬 구매 경험과 구매 이유

병원 처방 없이 호르몬 구입 경험	분포 (n=243)	성별정체성 범주[1]	
		트랜스여성 범주(n=160)	트랜스남성 범주(n=83)
	n (%)	n (%)	n (%)
없다	182 (74.9)	109 (68.1)	73 (88.0)
있다	61 (25.1)	51 (31.9)	10 (12.0)
병원 처방 없이 호르몬 구매 경로(n=61)[2]			
있다 - 처방이 필요 없는 다른 약물을 사용 (약국에서 피임약 구매 등)	28 (45.9)	26 (51.0)	2 (20.0)
있다 - 해외 온라인 판매자를 통해서 구매	19 (31.1)	18 (35.3)	1 (10.0)
있다 - 지인을 통해서 구매	15 (24.6)	8 (15.7)	7 (70.0)
있다 - 국내 온라인 판매자를 통해서 구매	10 (16.4)	9 (17.6)	1 (10.0)
있다 - 오프라인 판매자를 통해서 구매	5 (8.2)	4 (7.8)	1 (10.0)
병원 처방 없이 호르몬 구매 이유(n=61)[2]			
정신과 진단서가 없어서	34 (55.7)	28 (54.9)	6 (60.0)
호르몬제를 처방받을 수 있는 병원을 몰라서	15 (24.6)	11 (21.6)	4 (40.0)
의료적 조치에 대한 기록을 남기기 싫어서	10 (16.4)	8 (15.7)	2 (20.0)
비용이 더 저렴해서	10 (16.4)	6 (11.8)	4 (40.0)
병원에 가기 싫어서	10 (16.4)	6 (11.8)	4 (40.0)
기타	17 (27.9)	14 (27.5)	3 (30.0)

[1]성별정체성 범주는 출생 시 법적 성별이 남성인 트랜스여성 범주(트랜스여성와 논바이너리 남성)와 출생 시 법적 성별이 여성인 트랜스남성 범주(트랜스남성과 논바이너리 여성)을 포함한다.
[2]병원 처방 없이 호르몬을 구매한 경로와 구매한 이유는 중복 선택이 가능하다.

47.1%), “건강상의 이유 때문에”(n=23, 45.1%) 등을 선택하였다.

호르몬조치를 한 번이라도 경험한 적이 있는 트랜스젠더 참여자 총 243명 중 병원의 처방없이 호르몬을 구입해 본 참여자는 총 61명(25.1%)이었고, 트랜스여성 범주의 160명 중 51명(31.9%), 트랜스남성 범주의 83명 중 10명(12.0%)이 여기에 해당했다(표 6). 병원의 처방없이 호르몬을 구입한 참여자 61명에게 구입경로를 물어본 결과, 피임약과 같이 “처방이 필요 없는 다른 약물”(n=28, 45.9%)을 사용한 경우가 가장 큰 비중을 차지했다. 그 외에도 “해외 온라

표 7. 한국 트랜스젠더의 성전환수술 여부와 수술받지 않은 이유

성전환수술 여부	분포 (n=271)	성별정체성 범주[1]	
		트랜스여성 범주(n=168)	트랜스남성 범주(n=103)
	n (%)	n (%)	n (%)
받았다	115 (42.4)	57 (33.9)	58 (56.3)
받지 않았다	156 (57.6)	111 (66.1)	45 (43.7)
성전환수술을 받지 않은 이유(n=156)[2]			
비용이 부담되어서	122 (78.2)	85 (76.6)	37 (82.2)
나중에 수술을 받고자 하지만, 아직 수술을 받기 위한 준비가 되지 않아서	100 (64.1)	68 (61.3)	32 (71.1)
구직, 직장생활 등 경제적 활동에 어려움이 생길 것 같아서	57 (36.5)	37 (33.3)	20 (44.4)
가족, 지인의 반대로	39 (25.0)	30 (27.0)	9 (20.0)
수술 과정이 너무 위험해서	36 (23.1)	27 (24.3)	9 (20.0)
주변 사람들의 눈이 두려워서	32 (20.5)	22 (19.8)	10 (22.2)
수술에 필요한 의료적 요건을 맞추지 못해서	27 (17.3)	19 (17.1)	8 (17.8)
만족스러운 성기능을 가질 수 없어서	19 (12.2)	9 (8.1)	10 (22.2)
수술을 받을 수 있는 의료기관이 없어서	11 (7.1)	6 (5.4)	5 (11.1)
필요하지 않다고 생각하기 때문에	10 (6.4)	5 (4.5)	5 (11.1)
임신이나 출산 등 가족 계획이나 재생산에 문제가 생겨서	7 (4.5)	3 (2.7)	4 (8.9)
수술을 거부당해서	1 (0.6)	1 (0.9)	0 (0.0)
기타	13 (8.3)	10 (9.0)	3 (6.7)

[1]성별정체성 범주는 출생 시 법적 성별이 남성인 트랜스여성 범주(트랜스여성와 논바이너리 남성)와 출생 시 법적 성별이 여성인 트랜스남성 범주(트랜스남성과 논바이너리 여성)을 포함한다.
[2]성전환수술을 받지 않은 이유는 중복 선택이 가능하다.

인 판매자”(n=19, 31.1%), “지인”(n=15, 24.6%) 등을 통해 병원의 처방없이 호르몬을 구입한 것으로 나타났다. 참여자들은 병원의 처방없이 호르몬을 구입한 이유로 “정신과 진단서가 없어서”(n=34, 55.7%)를 가장 많이 꼽았다.

한 가지 이상의 성전환수술을 받았다고 응답한 115명을 성별정체성에 따라 구분하면, 168명의 트랜스여성 범주 중 57명(33.9%), 103명의 트랜스남성 범주 중 58명(56.3%)이었다(표 7). 성전환수술을 전혀 받지 않은 트랜스젠더는 156명(57.6%)이었으며, 이들에게 수술을 받지 않은 이유에 대해 물었다. 응답 중에는 “비용이 부담되어서”(n=122, 78.2%) 성전환수술을 받지 않았다는 응답이 가장 많았다. 성전환수술을 받지 않은 다른 이유로는 “나중에 수술을 받고자 하지만, 아직 수술을 받기 위한 준비가 되지 않아서”(n=100, 64.1%), “구직, 직장생활 등 경제적 활동에 어려움이 생길 것 같아서”(n=57, 36.5%) 등이 있었다.

논 의

본 연구는 한국에서 트랜스젠더의 의료적 트랜지션 관련 경험과 그 과정에서 장벽으로 작용하는 요인을 파악하기 위해 만 19세 이상의 성인 트랜스젠더 278명이 참여한 단면조사 자료를 분석하였다. 연구 결과, 전체 참여자 278명 중 91.0%인 253명이 성주체성장애 진단을 받았다. 호르몬요법 문항에 응답한 276명의 참여자 중 88.0%인 243명의 트랜스젠더가 호르몬투여 경험을 가지고 있었다. 성전환수술 여부를 묻는 문항에 응답한 참여자 271명 중 42.4%인 115명이 한 가지 이상의 성전환 관련 외과적 수술을 받은 적이 있다고 보고하였다.

참여자들이 의료적 트랜지션을 받지 않은 가장 큰 이유는 의료적 트랜지션 비용과 관련 있었다. 성주체성장애 진단을 받지 않은 25명 중 12명(48.0%)이 경제적 부담으로 인해 정신과진단을 받지 못했다고 응답하였다. 호르몬조치를 받지 않는 51명 중 28명(54.9%)과 성전환수술을 받지 않은 156명 중 122명(78.2%) 역시 의료적 트랜지션을 받지 않은 이유로 비용 부담을 언급했다. 이와 관련하여 트랜스젠더 참여자가 의료적 트랜지션에 지불했던 비용을 추가로 분석하였다(부록 표 1). 성주체성장애 진단을 위한 비용(n=251)의 경우, 가장 많은 설문참여자가 25-49만원(n=170, 67.7%)을 지출했다고 응답했다. 성전환수술은 가슴, 고환·정소/난소·자궁 제거, 성기 성형, 안면 성형, 성대(목소리) 성형, 기타수술로 구분하여 성별정체성에 따라 평균 소요비용을 계산하였다. 비용 부담이 가장 높은 수술은 성기 성형수술(트랜스여성 범주: 1,514.8만원, 트랜스남성 범주: 2,057.1만원)이었다.

의료적 트랜지션 비용과 더불어, 트랜스젠더에 대한 의료진의 편견과 차별적 태도 역시 트랜스젠더의 의료이용을 제한하는 요소인 것으로 나타났다. 이와 관련하여 본 연구에서 사용한 데이터를 활용해 의료인에게 진료를 거부당한 경험과 의료기관 방문을 회피하거나 연기한 경험을 추가 분석하였다(부록 표 2). 총 응답자 262명 중 지난 12개월 동안 스스로 의료기관 방문을 회피 또는 연

기한 적이 있다고 보고한 참여자는 112명(42.7%)이었으며, 의료기관 방문 시 의료진에게 진료를 거부당한 경험이 있는 참여자는 14명(5.3%)이었다. 2014년 성적지향·성별정체성에 따른 차별 실태조사에 따르면, 최근 5년간 의료기관을 이용한 적이 있는 트랜스젠더 78명 중 28명(35.9%)이 의료진에게 부적절한 질문이나 비난을 받는 등 의료기관에서 차별을 경험했다고 응답하였다[18].

트랜스젠더 참여자들이 겪을 수 있는 또 다른 장벽으로는, 의료적 트랜지션에 대한 의료진의 지식 부족과 의료적 조치를 받을 수 있는 의료기관 부족을 꼽을 수 있다. 정신과진단을 받지 않은 트랜스젠더 참여자 25명 중 일부는 "제대로 진단해 주는 정신과를 찾을 수 없어서"(n=7, 28.0%) 진단을 받지 않았다고 응답했다. 대부분의 경우 성주체성장애 진단을 받은 이후에 호르몬요법이나 성전환수술을 받을 수 있기 때문에, 성주체성 장애진단은 이후 의료적 트랜지션에 대한 접근을 결정하는 역할을 한다[19]. 실제로 참여자 중에는 "정신과진단이 없어서"(n=17, 33.3%) 호르몬요법을 받지 못하는 경우도 있었다. 호르몬조치를 제공하는 "의료기관이 없어서"(n=8, 15.7%) 호르몬을 투여하지 못하는 경우 역시 연구결과를 통해 확인할 수 있었다.

세계 트랜스젠더 보건의료 전문가 협회(The World Professional Association for Transgender Health, WPATH)는 트랜스젠더를 위한 건강관리실무표준(standards of care)을 통해 정신과진단 및 호르몬요법, 성전환수술 등에 대한 임상 지침을 제공하고 있다[2]. 북미나 유럽에서는 의료기관이나 학회를 중심으로 트랜스젠더의 의료적 트랜지션에 대한 교육과 수련과정을 개발하고 있다[7]. 나아가 이러한 교육과 수련 프로그램의 효과를 평가하기 위한 연구도 진행되고 있다[20,21]. 연구 결과에 따르면, 의료적 트랜지션에 대한 교육과 수련을 통해 의과대학 학생들은 트랜스젠더 건강에 대한 지식과 태도, 기술 측면에서 의료진으로서 역량을 강화할 수 있었다. 그러나, 한국의 의과대학 교육 및 수련과정에는 트랜스젠더의 의료적 트랜지션에 대한 내용이 포함되어 있지 않기 때문에 의료전문가의 의료적 트랜지션에 대한 지식은 낮은 수준에 머물러 있다[7].

트랜스젠더에 대한 부정적 사회인식 역시 참여자들이 의료적 트랜지션을 받지 않은 이유 중 하나로 보고되었다. 호르몬요법의 경우, "구직, 직장생활 등 경제적 활동에 어려움이 생길 것 같아서"(n=24, 47.1%), "주변 사람들의 눈이 두려워서"(n=13, 25.5%), "가족, 지인의 반대로"(n=8, 15.7%) 호르몬을 투여하지 않았다는 응답이 있었다. 성전환수술을 받지 않은 이들 역시 "구직, 직장생활 등 경제적 활동에 어려움이 생길 것 같아서"(n=57, 36.5%), "가족, 지인의 반대로"(n=39, 25.0%), "주변 사람들의 눈이 두려워서"(n=32, 20.5%) 수술을 받지 않았다고 응답했다. 트랜스젠더에 대한 인식이 낮은 사회환경 속에서 호르몬요법과 성전환수술 경험으로 인해 트랜스젠더는 취업을 하지 못하거나 직장생활을 지속하지 못해 경제활동에 어려움을 겪을 수 있고, 가족과 지인, 주변 사람들이 본인의 의료적 트랜지션을 반대할 수 있다. 이러한 요소들이 응답자로 하여금 호르몬조치나 성전환수술을 받지 못하게 하는 장벽으로 작용함을 확인하였다.

본 연구 결과, 트랜스젠더는 의료적 트랜지션을 받는 과정에서 높은 의료적 조치 비용으로 인한 경제적 부담, 의료기관에서의 차별이나 부정적 경험, 적절한 서비스를 제공하는 의료진과 의료시설의 부족, 트랜스젠더에 대한 낮은 사회인식 등 의료적 트랜지션 과정에서 다양한 장벽을 경험하고 있었다. 이러한 장벽은 트랜스젠더가 안전하지 않은 방법을 통해 의료적 트랜지션을 시도하게 만드는 요인이 될 수 있다[22,23]. 본 연구에 참여한 트랜스젠더 중 병원의 처방없이 호르몬을 구입한 경험이 있는 참여자는 243명 중 61명(25.1%)이었다. 이들은 피임약처럼 호르몬 성분을 포함하고 있으면서, 처방전 없이 구매 가능한 약물을 사용하거나 국내외 온라인 판매자 또는 지인을 통해 호르몬제를 구입하였다. 호르몬요법을 안전하게 받기 위해서는 개인의 건강상태에 따라 용량을 조절해야 하고, 이를 위한 정기검진이 중요하다. 호르몬 자가처방은 혈전색전증이나 간수치 상승 등 내과적 합병증이 발생했을 때 적절한 조치를 할 수 없다는 점에서 특히 위험할 수 있다[7,23].

트랜스젠더의 의료적 트랜지션에 대한 접근성을 향상하기 위해서는 무엇보다 호르몬요법 및 성전환수술에 대한 건강보험 적용이 필요하다. 이호림 등[7]에 따르면, 전 세계 118개국 중 국가건강보험이나 공공보건의료시스템을 통해 한 가지 이상의 의료적 트랜지션에 소요하는 비용을 부담하는 나라는 미국과 캐나다를 포함하여 총 45개국이다[7]. 한국은 현재 트랜스젠더의 호르몬요법과 성전환수술 비용 모두 공공보건의료시스템에서 보장하지 않고 있다. 의료적 트랜지션에 대한 건강보험 적용이 본 연구에서 드러난 의료적 트랜지션의 주된 장벽 중 하나인 경제적 비용 부담을 완화할 수 있는 정책적 대안이라는 점을 고려할 때, 의료적 트랜지션에 대한 건강보험 적용에 대해 보다 적극적인 학술적 논의가 필요하다.

본 연구의 주요한 한계는 설문조사에 참여한 트랜스젠더가 한국에 거주하는 트랜스젠더 인구집단을 대표하기 어렵다는 점이다. 2018년 현재까지 국내에서 트랜스젠더 인구 규모를 파악하기 위한 연구는 한번도 진행된 적 없다[7,16]. 이로 인해 본 연구는 한국에서 진행된 가장 큰 규모의 트랜스젠더 건강 연구(n=278)임에도 불구하고, 트랜스젠더 모집단의 규모를 알 수 없기 때문에 대표성에 있어 한계를 갖는다. 이러한 조건 속에서 연구팀은 트랜스젠더 인구집단에 최대한 접근하기 위해 의료기관과 퀴어문화축제, 온·오프라인 커뮤니티 등 여러 경로를 통해 트랜스젠더 설문 참여자를 모집하였다. 그러나 이러한 수집방법은 연구 결과에 영향을 끼칠 수 있다. 예를 들어, 전체 응답자 278명의 데이터 수집경로를 살펴보면, 의료기관을 통해 130명(46.8%)이 참여했으며, 그 다음

으로 온·오프라인 커뮤니티와 퀴어문화축제를 통해 각각 126명(45.3%)과 22명(7.9%)이 설문조사에 참가했다. 정신과진단과 호르몬요법의 경우, 의료기관을 통해 설문조사에 참여한 응답자들의 경험이 온·오프라인 커뮤니티와 퀴어문화축제를 통해 참여한 이들의 경험보다 상대적으로 많은 것으로 나타났다(표 3). 호르몬요법을 받기 위해서는 정신과진단이 필요한 경우가 많고, 호르몬을 투여하기 위해 정기적으로 의료기관을 방문해야 한다. 따라서 의료기관을 통해 참여한 이들의 정신과진단이나 호르몬조치 경험이 높게 보고될 수 있다. 그 뿐만 아니라 응답자의 연령이 20대(78.4%)에 편중된 것 역시 온라인 설문조사에 접근할 수 있는 트랜스젠더를 편의표집했다는 점에 기인할 수 있다. 따라서, 본 연구 결과를 해석할 때 유의할 필요가 있다.

위와 같은 한계에도 불구하고 본 연구는 다음과 같은 의의를 지닌다. 첫째, 본 연구의 분석자료인 설문조사는 의료기관, 퀴어문화축제, 온·오프라인 커뮤니티 등 가능한 방식을 최대한 활용하였고, 그 결과 국내에서 가장 많은 성인 트랜스젠더가 참여하였다. 둘째, 본 연구는 한국 성인 트랜스젠더의 의료적 트랜지션 경험과 그 과정에서의 겪은 장벽을 양적 데이터를 통해 파악한 최초의 학술 연구다. 향후 진행될 연구에서는 본 연구에서 살펴본 트랜스젠더의 의료적 트랜지션 관련 경험과 그 과정에서 겪은 장벽을 고려하여 한국 트랜스젠더의 건강에 대해 더욱 심층적인 연구가 진행되어야 한다.

결 론

정신과진단 및 호르몬요법, 성전환수술과 같은 의료적 트랜지션은 본인의 성별위화감을 완화시키고자 하는 트랜스젠더에게 필요한 조치이다. 그러나 이는 개인적 의미에 그치지 않는다. 이들에게 의료적 트랜지션은 자신이 원하는 성별을 공적 신분서류에 기재하기 위해 필요한 법률적 요건이자 사회적 차별로부터 본인을 지킬 수 있는 방식이며, 화장실과 같이 남녀 성별을 기준으로 분리된 공공시설에 접근하기 위한 방법이기도 하다[19]. 트랜스젠더의 의료적 트랜지션에 대한 접근성을 향상하기 위해서는 호르몬요법과 성전환수술 등 의료적 트랜지션에 대한 건강보험 적용이 필수적이다. 그 뿐만 아니라 적절한 의료적 트랜지션을 제공할 수 있도록 의료진에 대한 교육과 수련이 제도화되어야 하며, 트랜스젠더에 대한 사회적 인식을 높이고 이들에 대한 낙인과 차별을 줄이기 위한 법·제도적 조치가 적극적으로 도입되어야 한다. 학술적 영역에서는 트랜스젠더의 건강에 대한 연구와 논의의 활성화를 위해 국가수준의 설문조사에 성별정체성에 대한 문항을 포함해야 한다. 더 나아가, 한국 트랜스젠더의 의료적 트랜지션 경험과 장벽에 대한 본 연구결과를 바탕으로 트랜스젠더의 건강과 삶의 질 향상을 위한 정책적 개입이 시급히 이루어져야 한다.

감사의 글

본 연구는 다음 스토리펀딩 “트랜스젠더 건강 연구 시작합니다”에 참여해주신 분들의 후원을 통해 수행되었으며, 변화를 꿈꾸는 과학기술인 네트워크(Engineers and Scientists for Change, ESC) 크라우드펀딩 1호로 연구지원을 받았습니다. 설문지 구성 및 설문조사 홍보 과정에서 소중한 도움을 주신 네 곳의 의료기관 관계자 및 트랜스젠더 인권단체 조각보 활동가, 자문해주신 분들에게 감사의 말씀드립니다. 무엇보다도 온·오프라인에서 설문조사에 참여해주신 모든 분들께 진심으로 감사드립니다.

참고문헌

1. American Psychological Association. Answers to your questions: about transgender people, gender identity, and gender expression; 2011 [cited 2018 Feb 9]. Available from: http://www.apa.org/topics/lgbt/transgender.pdf.
2. Coleman E, Bockting W, Botzer M, Cohen-Kettenis P, DeCuypere G, Feldman J, et al. Standards of care for the health of transsexual, transgender, and gender-nonconforming people, version 7. Int J Transgend 2012;13:165-232.
3. James SE, Herman JL, Rankin S, Keisling M, Mottet L, Anafi M. The report of the 2015 U.S. transgender survey; 2016 [cited 2018 May 24]. Available from: https://www.transequality.org/sites/default/files/docs/USTS-Full-Report-FINAL.PDF.
4. Reisner SL, Poteat T, Keatley J, Cabral M, Mothopeng T, Dunham E, et al. Global health burden and needs of transgender populations: a review. Lancet 2016;388:412-436.
5. Meerwijk EL, Sevelius JM. Transgender population size in the United States: a meta-regression of population-based probability samples. Am J Public Health 2017;107:e1-e8.
6. Korean Statistical Information Service. KOSIS 100 indicators. [cited 2018 Feb 9]. Available from: http://kosis.kr/conts/nsportalStats/nsportalStats_0102Body.jsp?menuId=10&NUM=1014 (Korean).
7. Yi H, Lee H, Yoon JW, Park J, Kim S-S. Transgender people's access to health care in Korea. Health Soc Welf Rev 2015;35:64-94 (Korean).
8. Murad MH, Elamin MB, Garcia MZ, Mullan RJ, Murad A, Erwin PJ, et al. Hormonal therapy and sex reassignment: a systematic review and meta-analysis of quality of life and psychosocial outcomes. Clin Endocrinol (Oxf) 2010;72:214-231.
9. Weyers S, Elaut E, De Sutter P, Gerris J, T'sjoen G, Heylens G, et al. Long-term assessment of the physical, mental, and sexual health among transsexual women. J Sex Med 2009;6:752-760.
10. Wilson EC, Chen YH, Arayasirikul S, Wenzel C, Raymond HF. Connecting the dots: examining transgender women's utilization of transition-related medical care and associations with mental health, substance use, and HIV. J Urban Health 2015;92:182-192.
11. Son I, Lee H, Park J, Kim SS. Social stigma and medical marginalization in healthcare service among transgender people in South Korea. Korean J Sociol 2017;51:155-189 (Korean).
12. Choi SK. The sex correction of transsexuals and its standards: focusing on 'the Supreme Court 2011.9.2. 2009스 117' case. Korean J Fam Law 2013;27:373-412 (Korean).
13. Hong SP, Lee SH. A legal appraisal of the medical intervention in the

gender recognition of transgender under the international human rights and the Korean law, with focus on the recent decision of the Seoul Western District Court. Korean J Int Comp Law 2013; 58:131-160 (Korean).
14. Park H. Transgender people and their obligation to serve in the military in South Korea. In: The 8th LGBTI Human Rights Forum; 2016 Mar 4-6; Seoul, South Korea. Seoul: The 8th LGBTI Human Rights Forum Planning Group; 2016. p. 131-141 (Korean, author's translation).
15. Kim YJ. A study of fundamental rights that the person owns transsexualism is guaranteed [dissertation]. Seoul: Korea University; 2012 (Korean).
16. Lee H, Park J, Kim SS. LGBTQI health research in South Korea: a systematic review. Health Soc Sci 2014;36:43-76 (Korean).
17. Reisner SL, Conron KJ, Tardiff LA, Jarvi S, Gordon AR, Austin SB. Monitoring the health of transgender and other gender minority populations: validity of natal sex and gender identity survey items in a US national cohort of young adults. BMC Public Health 2014;14:1224.
18. Chang SY, Kim JH, Kim HK, Na YJ, Jung HH, Ryu MH, et al. A survey on discrimination based on sexual orientation and gender identity in South Korea; 2014 [cited 2018 May 24]. Available from: https://library.humanrights.go.kr/search/media/img/CAT000000038573?metsno=000000000936&fileid=M000000000936_FILE 000001 (Korean).
19. Dewey JM, Gesbeck MM. (Dys) Functional diagnosing mental health diagnosis, medicalization, and the making of transgender patients. Humanit Soc 2017;41:37-72.
20. Dowshen N, Nguyen GT, Gilbert K, Feiler A, Margo KL, Stroumsa D. Improving transgender health education for future doctors. Am J Public Health 2014;104:e5-e6.
21. Safer JD, Pearce EN. A simple curriculum content change increased medical student comfort with transgender medicine. Endocr Pract 2013;19: 633-637.
22. Lombardi E. Enhancing transgender health care. Am J Public Health 2001;91:869-872.
23. Rotondi NK, Bauer GR, Scanlon K, Kaay M, Travers R, Travers A. Nonprescribed hormone use and self-performed surgeries: "do-it-yourself" transitions in transgender communities in Ontario, Canada. Am J Public Health 2013;103:1830-1836.

부록 표 1. 한국 트랜스젠더의 성주체성장애 진단 비용과 성전환수술 비용

성주체성장애 진단 비용(n=251)	분포	성별정체성 범주[1]	
		트랜스여성 범주(n=162)	트랜스남성 범주(n=89)
	n (%)	n (%)	n (%)
24만원 이하	42 (16.7)	26 (16.0)	16 (18.0)
25만원-49만원	170 (67.7)	110 (67.9)	60 (67.4)
50만원-74만원	22 (8.8)	11 (6.8)	11 (12.4)
75만원 이상	17 (6.8)	15 (9.3)	2 (2.2)

성전환수술(n=115)	분포	트랜스여성 범주(n=57)		트랜스남성 범주(n=58)	
	n	n	평균 비용(표준편차)[1]	n	평균 비용(표준편차)[1]
가슴 수술	71	17	532.4 (443.3)	54	368.5 (146.4)
고환·정소/난소·자궁제거 수술	63	21	297.1 (523.8)	42	397.6 (290.1)
성기성형수술	34	27	1,514.8 (657.3)	7	2,057.1 (1,135.6)
안면성형수술	30	27	1,159.6 (1,048.5)	3	366.7 (57.7)
성대(목소리)성형수술	9	9	655.6 (545.7)	.	.
기타 수술	9	7	132.9 (118.1)	2	100 (0.0)

[1]성별정체성 범주는 출생 시 법적 성별이 남성인 트랜스여성 범주(트랜스여성와 논바이너리 남성)와 출생 시 법적 성별이 여성인 트랜스남성 범주(트랜스남성과 논바이너리 여성)을 포함한다.

부록 표 2. 한국 트랜스젠더의 인구사회학적 특성에 따른 지난 1년간 의료기관 방문 회피·연기 경험과 의료인으로부터 진료거부 경험

	분포 (n=262)	의료기관 방문을 회피하거나 연기한 적 있다(n=112)		의료인에게 진료를 거부당한 적 있다 (n=14)	
	n (%)	n (%)	p-value[1]	n (%)	p-value[2]
성별정체성 범주[3]			0.016		0.167
트랜스여성 범주	160 (61.1)	59 (36.9)		11 (6.9)	
트랜스남성 범주	102 (38.9)	53 (52.0)		3 (2.9)	
연령(세)			0.039		0.909
19-29	209 (79.8)	96 (45.9)		11 (5.3)	
30-50	53 (20.2)	16 (30.2)		3 (5.7)	
성적지향			0.273		0.003
이성애	123 (46.9)	52 (42.3)		4 (3.3)	
동성애	26 (9.9)	9 (34.6)		2 (7.7)	
양성애	90 (34.4)	37 (41.1)		3 (3.3)	
무성애	23 (8.8)	14 (60.9)		5 (21.7)	
교육 수준[4]			0.237		0.622
고등학교 졸업	57 (22.5)	25 (43.9)		2 (3.5)	
2년제 대학 재학·중퇴·졸업	39 (15.4)	12 (30.8)		2 (5.1)	
4년제 대학 재학·중퇴·졸업	144 (56.9)	68 (47.2)		10 (6.9)	
대학원 이상	13 (5.1)	4 (30.8)		0 (0.0)	
연평균 가구소득[4]			0.378		0.669
1,000만원 미만	69 (27.5)	30 (43.5)		6 (8.7)	
1,000만원-1,999만원	52 (20.7)	28 (53.8)		3 (5.8)	
2,000만원-2,999만원	45 (17.9)	16 (35.6)		1 (2.2)	
3,000만원-4,999만원	43 (17.1)	16 (37.2)		2 (4.7)	
5,000만원 이상	42 (16.7)	17 (40.5)		2 (4.8)	
고용 형태[4]			0.670		0.927
실업 또는 무직	117 (46.4)	53 (45.3)		7 (6.0)	
정규직	45 (17.9)	21 (46.7)		2 (4.4)	
비정규직	78 (31.0)	29 (37.2)		3 (3.8)	
자영업	11 (4.4)	5 (45.5)		1 (9.1)	
무급가족종사자	1 (0.4)	0 (0.0)		0 (0.0)	
거주 지역[4]			0.426		0.224
서울특별시	104 (40.9)	49 (47.1)		4 (3.8)	
광역시[5]	42 (16.5)	15 (35.7)		1 (2.4)	
시·군 지역	108 (42.5)	45 (41.7)		9 (8.3)	
설문조사 참여 경로			0.071		0.155
의료 기관	127 (48.5)	48 (37.8)		7 (5.5)	
퀴어문화축제(서울, 대구)	22 (8.4)	14 (63.6)		3 (13.6)	
온라인·오프라인 트랜스젠더 커뮤니티	113 (43.1)	50 (44.2)		4 (3.5)	

[1]인구사회학적 변수에 따른 지난 1년간 의료기관 방문 회피·연기 경험 빈도를 비교한 카이제곱 검정의 p-value.
[2]인구사회학적 변수에 따른 지난 1년간 의료진으로부터 진료거부 경험 빈도를 비교한 카이제곱 검정의 p-value.
[3]성별정체성 범주는 출생 시 법적 성별이 남성인 트랜스여성 범주(트랜스여성와 논바이너리 남성)와 출생 시 법적 성별이 여성인 트랜스남성 범주(트랜스남성과 논바이너리 여성)을 포함한다.
[4]각 항목별 무응답자 수는 다음과 같다: 교육 수준(n=9), 연평균 가구소득(n=11), 고용 형태(n=10), 거주지역(n=8).
[5]세종특별자치시를 포함한다.
